# iMAP: integration of multiple single-cell datasets by adversarial paired transfer networks

**DOI:** 10.1186/s13059-021-02280-8

**Published:** 2021-02-18

**Authors:** Dongfang Wang, Siyu Hou, Lei Zhang, Xiliang Wang, Baolin Liu, Zemin Zhang

**Affiliations:** 1 BIOPIC and School of Life Sciences, Peking University, Beijing, China; 2 MOE Key Laboratory for Bioinformatics, BNRIST Bioinformatics Division, Department of Automation, Tsinghua University, Beijing, China; 3 Institute of Cancer Research, Shenzhen Bay Laboratory, Shenzhen, China; 4 Analytical Biosciences Limited, Beijing, China; 5 Beijing Advanced Innovation Center for Genomics, Peking-Tsinghua Center for Life Sciences, Peking University, Beijing, China

**Keywords:** scRNA-seq, Data integration, Deep learning, GAN

## Abstract

The integration of single-cell RNA-sequencing datasets from multiple sources is critical for deciphering cell-to-cell heterogeneities and interactions in complex biological systems. We present a novel unsupervised batch effect removal framework, called iMAP, based on both deep autoencoders and generative adversarial networks. Compared with current methods, iMAP shows superior, robust, and scalable performance in terms of both reliably detecting the batch-specific cells and effectively mixing distributions of the batch-shared cell types. Applying iMAP to tumor microenvironment datasets from two platforms, Smart-seq2 and 10x Genomics, we find that iMAP can leverage the powers of both platforms to discover novel cell-cell interactions.

**Supplementary Information:**

The online version contains supplementary material available at 10.1186/s13059-021-02280-8.

## Background

Single-cell RNA-sequencing (scRNA-seq) technologies have profoundly changed our understandings of cell-to-cell heterogeneities in various biological areas [1–3]. Compared with individual scRNA-seq experiments, integration of datasets from multiple sources can enlighten researchers on more reliable novel discoveries. However, inherent technical differences among experiments may lead to inescapable batch effects, confounding the biological variations [4–6]. Eliminating the unwanted technical variations among different datasets, but not diminishing those biological differences, is one major challenge for batch effect removal methodologies.

A number of unsupervised batch effect removal methods for scRNA-seq datasets have been developed in recent years, including a class that attempts to model the global relationships between batch effects and gene expression profiles. For examples, Combat [7] models the gene expressions as a function of batch origins, and LIGER [8] extracts the batch-specific gene factors from the whole expression profiles. Another class of methods is pioneered by the innovative idea of mutual nearest neighbors (MNNs) [9] between different batches, with paired cells used as local anchors to help batch correction of their neighborhood. BBKNN [10], Scanorama [11], and Seurat v3 [12] follow this idea but search the MNNs in elaborated dimension-reduced spaces, instead of the original expression vectors. Harmony [13] deploys a novel local correction idea that preferentially clusters cells from different batches, thereby better matching the distributions of the shared cell types across datasets. In theory, the former global correction methods may be beneficial to retain the dataset-specific biological variations, but do not fully guarantee the integration of the shared cell types. The performance of latter local corrections highly depends on the qualities of MNNs or matched local clusters. This makes it hard to balance between the identification of the dataset-specific cells and the mixture of the shared cell types. To address this major hurdle, here we develop a new framework to take advantages of both two strategies while overcoming the intrinsic challenges of them.

Our method, called iMAP—Integration of Multiple single-cell datasets by Adversarial Paired-style transfer networks—is a deep learning-based framework for batch effect removal of scRNA-seq datasets. Some studies such as scVI [14] and DESC [15] have showed the potentials of deep networks, especially the autoencoder structures, on processing scRNA-seq data, but autoencoder-based models usually have difficulties in reconstructing the batch-corrected transcriptomes with high fidelity. iMAP combines the powers of two kinds of state-of-art unsupervised deep network structures—autoencoders and generative adversarial networks (GANs) [16]. A novel autoencoder structure is used to build low-dimensional representations of the biological contents of cells disentangled from the technical variations. Then GANs are leveraged to remove the batch effects from the original expression profiles. Compared with other methods, iMAP could both match the distributions of the shared cell types and discern the batch-specific cell types on the benchmark datasets. We also demonstrate the stability of iMAP over the choice of hyperparameters and the effect of stochasticity and provide a framework to interpret the working mechanisms of iMAP. iMAP is scalable on large datasets with the notable speed advantage especially for datasets with cells more than thousands. Finally, we applied iMAP to the integration of tumor-infiltrating immune cell datasets sequenced by Smart-seq2 and 10x Genomics (10x) and discovered novel cell-cell interactions by virtue of the powers of both platforms. iMAP is available as a Python package on github (https://github.com/Svvord/iMAP).

## Results

### Overview of the iMAP algorithm

**Fig. 1:**
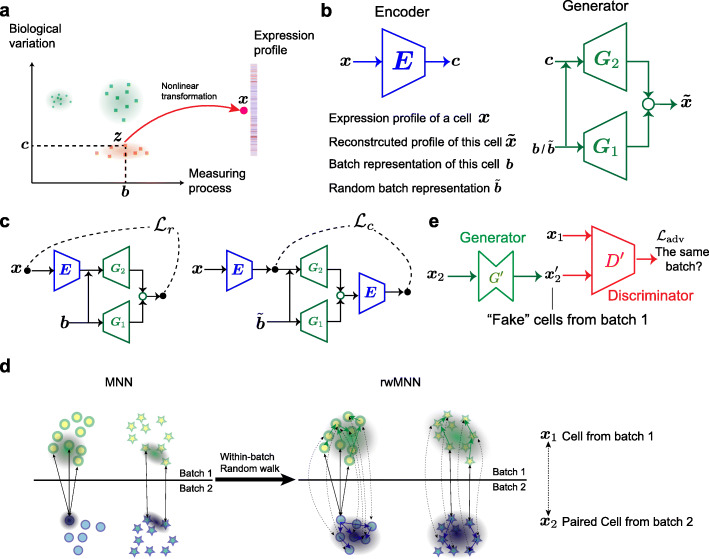
Overview of the iMAP algorithm. **a** The expression profiles modeled by a function of two independent factors: biological variations and measuring processes. **b** Three feed-forward neural networks deployed for the first stage of iMAP. **c** Information flows and two losses functions used in the first stage. *L*_*r*_, reconstruction loss; *L*_*c*_, content loss. **d** A within-batch random walk-based procedure adopted to extend the MNN pairs. These extended MNN pairs are called rwMNN pairs. **e** A GAN structure used to remove the batch effects based on the rwMNN pairs

Our iMAP framework consists of two stages, including building the batch-ignorant representations for all cells, and then guiding the batch effect removal of the original high-dimensional expression profiles (the “Methods” section). We model the measured expression profile *x* of one single cell as a function of two independent factors: the biological variation and measuring process (Fig. 1a), whose effects would be disentangled by an autoencoder-like deep neural network structure deployed in the stage I. This structure includes three feed-forward multi-layer neural networks: one encoder *E*, extracting low-dimensional representations of biological contents *c* from a cell’s expression profile *x*, and two generators *G*_1_, *G*_2_, reconstructing the expression profile from *c* and one batch indicator (Fig. 1b). If we feed the generators with the true batch indicator *b* of one cell, they could reconstruct the original expression profile. By contrast, if a random batch indicator $\overset{\sim }{b}$ is inputted, the generators should fabricate a pseudo-cell but with the same biological content as the original true cell. This inspired two loss functions: the reconstruction loss *L*_*r*_ and the content loss *L*_*c*_ (Fig. 1c). After training, we expect the encoder to capture batch-ignorant representations from the single-cell transcriptomes. By virtue of these representations and the adversarial networks, we could further remove the batch effects on the original high-dimensional expression profiles in the stage II, using a similar strategy as the pair-based neural style transfer in the computer vision field [17]. iMAP extracts the MNN pairs from two batches using the representations obtained from the previous stage, increasingly resulting in much larger number of high-quality MNN pairs compared with that using the original expression profiles. One potential problem of MNN pairs is that they may not fully cover the whole distributions of the shared cell types between two batches. Therefore, iMAP regards the cells in the MNN pairs as initial seeds, and adopts a random walk-based method to enroll new pairs, through successively selecting a cell from the kNNs (*k* nearest neighbors) of the seeds within each batch (Fig. 1d). These extended pairs are called rwMNN pairs. A GAN-based structure, composed of one generator *G*^′^ and one discriminator *D*^′^, is then trained only on these rwMNN pairs (Fig. 1e), while all cells could be transformed to remove the batch effects using the trained generator. We argue that the rwMNN pair is of vital importance for the GAN to correctly match the complete cell expression distributions of two batches. In the case of multiple batches, an incremental batch effect removal process is used (the “Methods” section).

### Benchmark evaluations

We first adopted two publicly available well-controlled datasets to qualitatively and quantitatively evaluate the performance of iMAP, in terms of both well-mixing the distributions of the shared cell types between different batches and identifying those batch-specific cells. Current evaluation metrics of batch effect removal can be classified as cluster-level and single cell-level indices, where the former ones, including ASW (Average silhouette width) and ARI (Adjusted rand index), are easy to compute but cannot reliably evaluate the mixture of cells from different batches within the local neighborhood (Additional file 1: Fig. S1a). Therefore, we focused on the single cell-level metrics. One famous single cell-level metric, kBET (k-Nearest neighbor batch-effect test) [18] assesses the batch mixing by comparing the batch distribution within kNNs of a cell with the global batch distribution, but it ignores the diversity of cell-type proportions of different batches [13]. Another single cell-level metric, LISI (Local Inverse Simpson’s Index) [13], overcomes the above difficulties and evaluates the mixing of batches and separation of cell types using two indices, i.e., iLISI (integration LISI) and cLISI (cell-type LISI). The possible drawback of LISI is that it is hard to summarize all single cell-level LISI values into a simple statistic for comparing between various methods. We then devised a novel evaluation procedure at the single-cell level, to qualitatively visualize and quantitatively summarize the performance of batch effect removal methods on both the effectiveness of mixing the shared cell types and discerning the batch-specific cell types (the “Methods” section; Additional file 1: Fig. S1b). This procedure includes two local classifiers for each single cell, the first of which would discriminate those cells surrounded by others with the same cell type as “positive” and otherwise “negative.” The second classifier would further pick out the “true positive” cells from those positive ones. The “true positive” cells are the cells which have congruous local batch distribution with the global batch distribution of its cell type. The proportions of positive cells and true positive cells can be used as the summary metrics to quantitatively compare overall performance of batch effect removal methods.

**Fig. 2:**
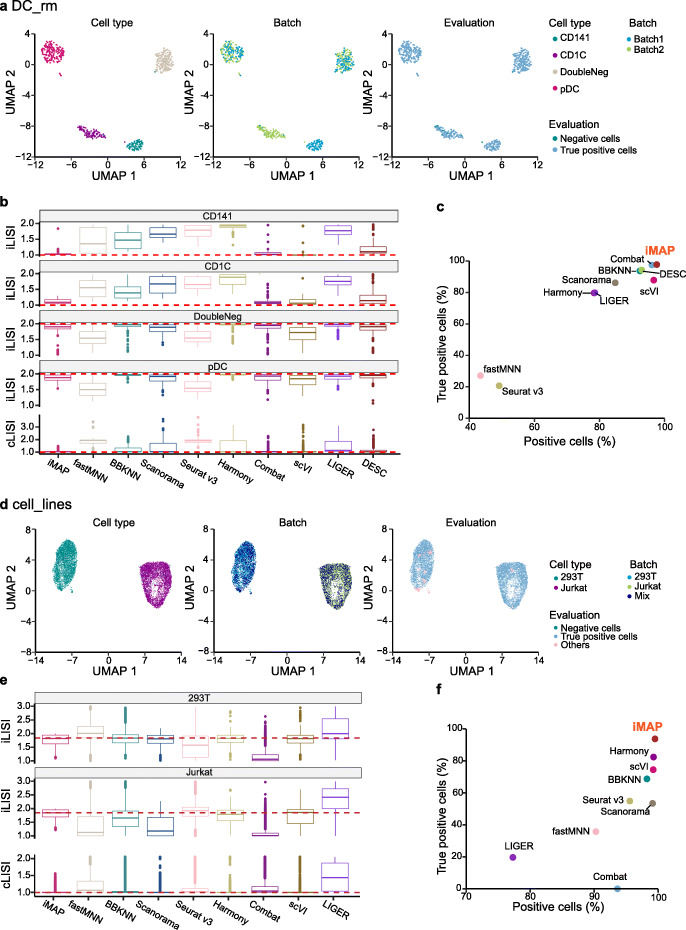
Benchmarks of batch effect removal methods. **a** Visualizations of iMAP batch effect removal results on the “DC_rm” dataset. Three kinds of colors are used to illustrate the cell type, batch, and evaluation information. **b** iLISI values for two cell types and cLISI values obtained by different methods on the “DC_rm” datasets. The dashed lines (the same below) represent the best theoretical LISI values. **c** Quantitative assessments of different batch effect removal methods on the “DC_rm” dataset. Two indices—proportion of positive cells (surrounded by cells with the same cell type) and proportion of true positive cells (surrounded by appropriate fractions of cells from different batches)—are adopted. **d** Visualizations of iMAP batch effect removal results on the “cell_lines” dataset. **e** Distributions of LISI values on the “cell_lines” dataset. **f** Quantitative assessments of different batch effect removal methods on the “cell_lines” dataset. See also Additional file 1: Fig. S2, 3, 4, 5

Our first benchmark dataset is composed of two batches, both sequenced using the Smart-seq2 protocol, and consists of four kinds of human dendritic cells (DCs), i.e., CD1C DC, CD141 DC, plasmacytoid DC (pDC), and double negative cells (DoubleNeg) [19] (Additional file 1: Table S1). Two types of biologically similar cells, CD1C DC from batch1 and CD141 DC from batch2, were removed to ensure the two sub-datasets contained batch-specific cells [6] (See Additional file 1: Fig. S2a for the complete “DC” dataset). So, we named the processed dataset as “DC_rm.” iMAP clearly separates the two batch-specific cell types, and well-integrates other batch-shared cell types (Fig. 2a). We also performed batch effect removals and visualizations using nine leading batch effect removal methods, including Combat, LIGER, fastMNN, BBKNN, Harmony, Scanorama, Seurat v3, scVI, and DESC (Additional file 1: Table S2; the “Methods” section). And it becomes challenging for some MNN pairs-based methods, such as Seurat v3, fastMNN, Harmony, Scanorama, and BBKNN to reliably discriminate these two biologically similar but batch-specific cell types. In contrast, iMAP, Combat, scVI, and DESC could clearly identify and separate them from others, although the former two methods perform much better integration of two batches (Additional file 1: Fig. S3). Quantitative analyses show that only iMAP and Combat give all LISI values closer to the best theoretical values (Fig. 2b), and the proportions of true positive cells of them (iMAP: 97.6%; Combat: 97.4%) are larger than other methods (Fig. 2c). This demonstrates that iMAP could effectively identify the batch-specific but biologically similar cell types.

The second dataset consists of three sub-datasets, including the “Jurkat” and “293 T” composed of cells from pure cell lines, and the “Mix” which is a 50/50 mix of cells from those two cell lines (Additional file 1: Table S1). We named this dataset as “cell_lines.” iMAP perfectly divides all cells into two components, each including one type of cells, and within each component, cells from two batches, i.e., “Jurkat/Mix” or “293 T/Mix,” are well-mixed (Fig. 2d). We also performed batch effect removals and visualizations using other batch effect removal methods (DESC was not compared because it was not optimized for processing datasets containing pure cell types) and compared their performances with iMAP. All methods except Combat could discriminate these two cell types, but with various separation capacities. fastMNN, LIGER, and Seurat v3 show inferior division of two cell types. For the integration power, iMAP and Harmony are notably stronger than others, while iMAP makes even better mixture than Harmony (Additional file 1: Fig. S4). Quantitative metrics show that iMAP performs the best on this dataset. cLISI values and iLISI values of iMAP are much closer to the best theoretical lines (Fig. 2e). The proportion of true positive cells of iMAP is 94.2%, and the next best value obtained by Harmony is 82.8%. All others give values less than 75% (Fig. 2f). We also adopted kBET to evaluate the integration of batch-shared cell types, and iMAP also gives the best performance (the “Methods” section; Additional file 1: Fig. S5). These demonstrate the ability of iMAP to match the distributions of the same cell type from different batches.

### Integration of human pancreas datasets

**Fig. 3:**
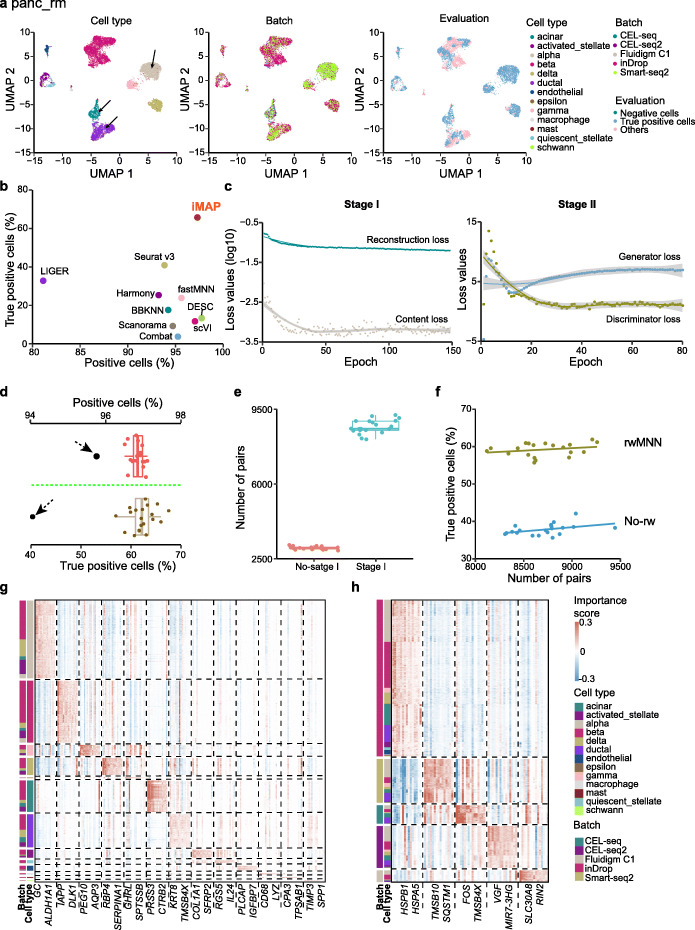
Integration of human pancreas datasets. **a** Visualizations of iMAP batch effect removal results on the “panc_rm” dataset. Three kinds of colors are used to illustrate the cell type, batch, and evaluation information. **b** Quantitative assessments of different batch effect removal methods on the “panc_rm” dataset. See also Additional file 1: Fig. S2, 5, 6. **c** The training process of the “panc_rm” dataset. The changes of loss values versus epochs for all four loss functions of two stages are shown. **d** Running stability of iMAP. We re-ran the iMAP on the “panc_rm” dataset for 20 times, and computed the proportions of positive and true positive cells. The marked black points are the best results obtained by other methods. **e** Stage I increasing the number of MNN pairs. **f** The rwMNN procedure boosting the proportion of true positive cells. The “Smart-seq2” and “inDrop” sub-datasets of the “panc” dataset were used to compare the number of MNN pairs. See also Additional file 1: Fig. S11. **g** Importance scores of selected genes for building the representations of each cell in stage I. **h** Importance scores of selected genes for removing batch effects in stage II

Next, we used iMAP to integrate human pancreas cells sequenced by different platforms, further assessing its performance and exploring its algorithmic properties. The whole dataset, named as “panc,” contains five sub-datasets, including “inDrop,” “CEL-seq,” “CEL-seq2,” “Smart-seq2,” and “Fluidigm C1,” indicating the characteristic sequencing protocols they used [20–24] (Additional file 1: Table S1; the “Methods” section). We still removed fractions of cells from some original datasets to ensure that certain batches included batch-specific cell types, for evaluating both the mixture of the distributions of the shared cell types between different batches and identification of those batch-specific cells. Particularly, we removed cells of two cell types with very large number of cells (i.e., “acinar” and “alpha” cells) from the “inDrop” sub-dataset, and “ductal” cells from the “CEL-seq” sub-dataset (See Additional file 1: Fig. S2b for the complete “panc” dataset; see the “Methods” section for the exclusion criteria). The updated dataset was named as “panc_rm.” We then performed integration using iMAP and all nine benchmark methods, comparing their performances. As shown in Fig. 3a, after integration by iMAP, the “acinar” and “alpha” cells are barely mixed with other cells from “inDrop,” and so are the “ductal” cells for “CEL-seq.” At the same time, all cell types are almost perfectly separated from others and the shared cell types from different platforms are well-mixed with each other. However, Harmony, LIGER, and Seurat v3 improperly mix one or more of “acinar,” “alpha,” and “ductal” cells with other cell types, while Combat, Scanorama, fastMNN, BBKNN, scVI, and DESC identify these batch-specific cell types, but with very limited integration of those shared cell types (Additional file 1: Fig. S6). Quantitative evaluation also indicates iMAP shows superior performance over all other methods, with 65.8% cells classified as true positive cells, whereas none of others could obtain over 50% of true positive cells (the next best method Seurat v3 has 40.9% true positive cells) (Fig. 3b). This demonstrates that iMAP can successfully integrate cells from multiple platforms with various numbers of cells and diverse compositions of cell types.

In summary, on all three benchmark datasets, iMAP shows consistently better performance over all other methods in terms of both the identification of the batch-specific, even biologically similar cell types, and the integration of the shared cell types across multiple batches. To further demonstrate the robustness of iMAP, we additionally used iMAP and all other nine batch effect removal methods on two recently published benchmark datasets [25], which sequenced thousands of cells from peripheral blood mononuclear cells and brain tissue respectively, with over ten protocols, covering most of single-cell and/or single-nucleus profiling methods (the “Methods” section; Additional file 1: Table S1). iMAP could still provide solid performance on these two complicated datasets (Additional file 1: Fig. S7). We also applied iMAP to remove batch effects on five additional datasets with various numbers of cells, various numbers of batches, and different sequencing platforms, where iMAP can all give solid integration performance (Additional file 1: Fig. S8; Additional file 1: Fig. S9; Additional file 1: Table S1).

### The stability and interpretability of iMAP

We further used the “panc_rm” dataset to demonstrate the stability of iMAP under the effects of hyperparameters and stochasticity. iMAP as a deep learning-based framework involves multiple hyperparameters necessary to be exploited for obtaining the optimal performance for specific dataset, a critical one of which is the number of training epochs. We examined the training process of the two stages of iMAP on the “panc_rm” dataset (Fig. 3c), observing a sharp decrease of loss values during the first epochs of stage I, which indicates that the network quickly reconstructed the expression profiles and extracted the low-dimensional embeddings of biological contents. After about 50 epochs, the content loss displayed very limited fluctuations. The training for stage II was much harder, especially for the generator. In the beginning the generator loss showed large vibration, while all losses tended to be stable after about 50 epochs. We conclude that the performance of iMAP may have limited changes if the number of training epochs is about 50–200 for both two stages. iMAP is also robust to changes in other hyperparameters (Additional file 1: Fig. S10; Additional file 1: Table S3). Then, we sought to observe the effects of intrinsic stochasticity of iMAP on the performance by re-running the entire iMAP procedure for 20 times on the “panc_rm” dataset. As expected, there existed inevitable fluctuations for both the proportions of positive and true positive cells, although the lower bounds were much higher than the best results obtained by other methods (Fig. 3d).

As we claimed before, the representations built by stage I would significantly increase the number of MNN pairs between two batches and the rwMNN procedure would exert huge influence over pair-based batch effect removal in the stage II and the final integration results. We first compared the number of pairs between the “inDrop” and “Smart-seq2” sub-datasets using the original expression profiles with that obtained by representations from stage I. The number of MNN pairs obtained after stage I (with the median 8610 over 20 repeat runs) was much higher than that obtained without stage I (median = 3034) (Fig. 3e), showing representations built by stage I of cells with the same cell type but from different batches are more similar than the original expression profiles of them. We then inspected the effects of rwMNN, observing that the proportion of true positive cells was sharply decreased after eliminating the random walk procedures from stage II (the median value over 20 repeat runs was decreased from 59.5 to 37.6%) (Fig. 3f). This indicates that rwMNN can better sketch the distribution of cells and assist GAN in capturing and removing the batch effects (see also Additional file 1: Table S4 and Additional file 1: Fig. S11).

Finally, we tried to interpret the working mechanisms of our neural networks through assigning importance scores for each gene, to evaluate its impacts on building the representations and removing the batch effects of each cell [26] (the “Methods” section; Additional file 1: Fig. S1c, d). The results showed that for the representations built by the encoder in the stage I, the most important genes were usually cell type-specific, and batch-neutral (Fig. 3g). For example, the gene *GC* was uniquely critical for representations of “alpha” cells, and we observed consistent importance scores across all five platforms (median values for “inDrop,” “Smart-seq2,” “CEL-seq,” “CEL-seq2,” and “Fluidigm C1” were 0.095, 0.095, 0.068, 0.076, and 0.098 respectively). In contrast, the most important genes for batch effect removal of stage II were mainly determined by batches, and most of them showed similar effects across all cell types (Fig. 3h). In summary, we provide a simple procedure to interpret our deep learning-based model and further prove our two-stage frameworks could convincingly remove the batch effects of scRNA-seq datasets.

### Application of iMAP on large-scale datasets

**Fig. 4:**
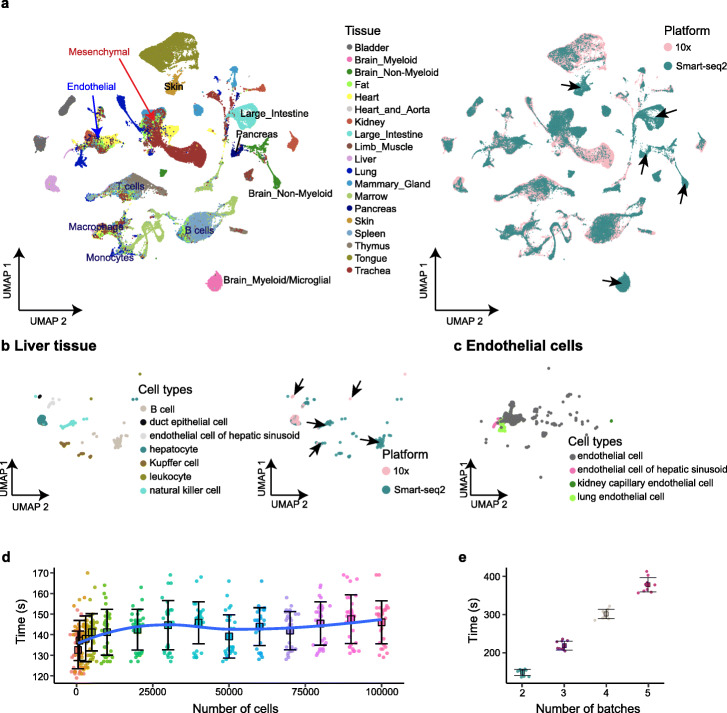
Integration of the large-scale Tabula Muris dataset. **a** Visualizations of iMAP batch effect removal results on the Tabula Muris dataset. Platform-specific tissues and tissue-shared cell types are marked. **b** Visualizations of cells from liver (subsets of **a**). Platform-specific cells are marked. **c** Visualizations of endothelial cells (subsets of **a**). **d**, **e** Time performance of iMAP. We ran iMAP multiple times using downsampled cells from Tabula Muris, to test the time cost versus the number of cells (**d**) and the number of batches (**e**). See also Additional file 1: Fig. S12

To demonstrate iMAP’s scalability on datasets with a large number of cells, we ran iMAP on the Tabula Muris dataset [27], containing over 100,000 cells, each sequenced by the Smart-seq2 or 10x platform. iMAP could both reliably integrate cells from the same tissues but sequenced by separate platforms and identify cells from platform-specific tissues, such as brain, large intestine, skin, and pancreas, which were exclusively obtained by Smart-seq2 (Fig. 4a; Additional file 1: Fig. S12a). We further confirmed the integration power of iMAP by exploiting the cell types mixture within individual tissues. For example, in the liver tissue, overall seven distinctive cell types were captured by these two platforms, including five platform-specific cell types, i.e., B cell (Smart-seq2), duct epithelial cell (10x), Kupffer cell (Smart-seq2), leukocyte (10x), and natural killer cell (Smart-seq2) (Additional file 1: Fig. S12b). We observed that the above five platform-specific cell types were well separated, and platform-shared cell types, i.e., endothelial cell of hepatic sinusoid and hepatocyte, were integrated together (Fig. 4b). Besides the tissue-specific cells, we also noticed some tissue-shared endothelial cells, mesenchymal cells, and immune cells, indicating their great biological similarities across tissues (Fig. 4a). However, iMAP still recognized distinctive endothelial cells from hepatic sinusoid, lung, and kidney capillary (Fig. 4c), indicating the particular functions of these types of cells in biological processes, such as cancer metastasis [28, 29].

Then, we evaluated the time cost of iMAP versus the number of cells by downsampling from 500 to 100,000 cells of Tabula Muris (Fig. 4d; Additional file 1: Fig. S12c). Initially, the time cost increased linearly with respect to the number of cells. As the number exceeded about thousands, the running time kept approximately constant, considering the inescapable instabilities of machines. We further used iMAP to integrate two datasets with 320,642 cord blood and 335,616 bone marrow-derived cells from the Human Cell Atlas, and iMAP can effectively remove the batch effects in a few minutes on a standard Linux server (Additional file 1: Fig. S12d, e; Additional file 1: Table S1; the “Methods” section). Finally, we simulated the effects of the number of batches on time costs. As shown in Fig. 4e, the running time increased linearly as the number of batches increased (the “Methods” section). In summary, iMAP could scale to large datasets with great integration powers and minimal time increasement with respect to the number of cells.

### iMAP identified mixed immune cell subsets and underappreciated interactions

**Fig. 5:**
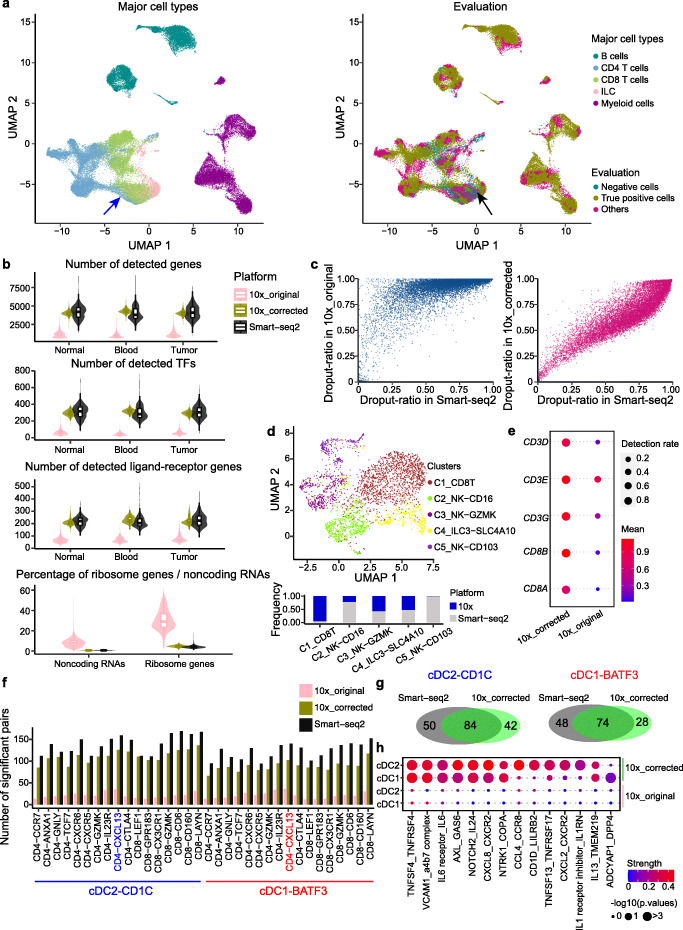
Applications of iMAP on CRC tumor-infiltrating immune cells. **a** Visualizations of major cell types and integration performance of iMAP. **b** Comparisons of the number of detected genes, transcription factors (TFs), ligand-receptor genes, and the percent of ribosome genes, noncoding RNAs between original 10x data, corrected 10x data, and Smart-seq2 data. **c** Comparisons of the dropout-ratio distribution between original 10x data, corrected 10x data, and Smart-seq2 data. **d** Re-analyzed of ILC subsets after batch effect removal. We showed new defined clusters and platform distribution over clusters. **e** New refined CD8^+^ T cell cluster with expression of key markers compared between original and corrected 10x data. **f** Significant ligand-receptor pairs (*p* < 0.05) defined between cDCs and T cells. **g** Significant ligand-receptor pairs detected between cDCs and Th1-like cells (CD4-CXCL13) by Smart-seq2 and corrected 10x data. **h** Selected specific pairs identified between cDCs and Th1-like cells by corrected 10x data. See also Additional file 1: Fig. S13

To examine the ability of iMAP to generate new biological insights, we applied iMAP on a scRNA-seq dataset of tumor-infiltrating immune cells from colorectal cancer (CRC) [30], which provides single-cell transcriptomes of over 50,000 immune cells from 18 CRC patients using both Smart-seq2 and 10x platforms. iMAP was adopted to remove the batch effects between two platforms and across different patients (the “Methods” section). The major cell types from both platforms were mostly correctly separated and well-integrated as shown by the UMAP embeddings [31] plot (Fig. 5a, Additional file 1: Fig. 13a). Of note, a small number of CD4^+^ effector T cells (CD4-GNLY) were located close to CD8^+^ effector T cells (CD8-CX3CR1), mainly due to their similarly high expression of cytotoxicity-related genes (such as *NKG7*, *GNLY*), and their same tissue origin of blood (Additional file 1: Fig. S13a, b).

Given that we can obtain the whole corrected transcriptomes of cells sequenced by 10x, it is possible to compare data from Smart-seq2 with original 10x data and batch-corrected 10x data on a global scale. As described previously, the number of detected genes for each cell obtained by 10x is significantly lower than that obtained by Smart-seq2 [30, 32]. However, after iMAP correction, we recovered the dropout genes by 10x and boosted the number to be almost at the same level as Smart-seq2 (Fig. 5b). For example, the median number of detected genes across cells from tumor was 3994 for corrected 10x, which was close to the median number 4085 for Smart-seq2. Particularly, the detected number of transcription factors and ligand-receptors were also promoted, which may facilitate further analyses of regulatory and cell-cell interactions (Fig. 5b). We next systematically checked the dropout-ratio of each gene across all cells in each platform. As expected, the relationship between dropout-ratios of genes acquired by 10x and Smart-seq2 was strongly upper convex. Matching the distribution of gene expression by iMAP made the dropout-ratios between batches much more consistent (Pearson’s correlation = 0.9) (Fig. 5c). Besides recovering the specific dropout genes by 10x, our method could also appropriately decrease the percentage of ribosome genes and noncoding RNAs (Fig. 5b), which usually comprise a large number of the whole sequenced transcriptomes obtained by 10x [32]. It is worth noting that the corrected 10x data were not just the same as that of Smart-seq2. For example, the variance of the number of detected genes was similar to the original 10x data, both smaller than that of Smart-seq2 (Fig. 5b). This may indicate iMAP could match the distribution of 10x and Smart-seq2 on average, but does not press to match each single cell.

For specific cell subsets, we noticed certain previously annotated innate lymphoid cells (ILCs), especially the NK-CD16 cells, from 10x were mixed with CD8^+^ effector cells after batch correction (Fig. 5a; Additional file 1: Fig. S13b). Such mixture could be caused by the high dropout-ratio of key marker genes for major cell types sequenced by 10x, such as *CD8A*, *CD8B* for CD8^+^ T cells, and *CD4* for CD4^+^ T cells (Additional file 1: Fig. S13c). Another possibility could be the functional similarity of CD16^+^ NK cells to their T and NKT counterparts, with the main transcriptomic distinctiveness of these NK cells being devoid of expression of T cell receptors and its associated adapters and co-receptors [33]. To better characterize ILCs, we re-clustered the defined ILCs from the original publication using batch effect-removed data and identified overall 5 clusters (Fig. 5d). The C3_NK-GZMK, C4_ILC3_SLC4A10, and C5-NK_CD103 were nearly identical to original annotations, and cells from 10x and Smart-seq2 were appropriately integrated (Additional file 1: Fig. S13d). However, the original NK-CD16 cells were clearly divided into two clusters, with one of them, C1_CD8T, showing significantly high expression of *CD8A*, *CD8B*, *CD3D*, *CD3E*, and *CD3G* (Fig. 5e). We further found that this cluster was 10x-specific and mainly from the blood, and in the original 10x data, these genes had already been detected sporadically (Fig. 5e). Therefore, it was reasonable to assume these pre-defined ILCs may be CD8^+^ effector T cells, although further evidence, such as TCR information should be considered. Considering that NK cells have attracted many research interests in recent years because of its enormous potentials for cancer immunotherapy [34–36], it should be careful to discern them from CD8^+^ effector T cells, especially with droplet-based sequencing technologies.

Finally, we explored novel cell-cell interactions uncovered by corrected 10x data. We focused on the interactions between DCs and T cells, because of the central roles of DCs in the cell-cell interaction network of the CRC tumor microenvironment [30]. We found that 10x could detect a comparable number of significant ligand-receptor pairs between cDC subsets (cDC2-CD1C and cDC1-BATF3) and various kinds of T cell subsets after batch effect removal (the “Methods” section; Fig. 5f). Among CD4^+^ T cells, the Th1-like cells (CD4-CXCL13) had the largest number of interaction pairs with both cDC2 and cDC1. Given the pivotal role of these cells in the CRC microenvironment and immunotherapies [37], we further dissected the predicted ligand-receptor pairs between cDCs and Th1-like cells. We found a high overlap of significant pairs between cDCs and Th1-like cells identified by Smart-seq2 and corrected 10x data (Fig. 5g). However, many specific interaction pairs were only captured by corrected 10x data, possibly because of the larger number of cells and distinctive proportions of cell types. For example, cDCs could secrete chemokines, such as *CXCL8*, to recruit Th1-like cells into tumor with the bonding of *CXCR2*. Additionally, the co-stimulatory ligand *TNFSF4* (OX40L) and its receptor *TNFRSF4* (OX40) were also highly expressed in cDCs and Th1-like cells, respectively. Their significant interaction may play an essential role in Th1-like cell activations in the CRC microenvironment [38], which may impact the anti-CD40 agonist treatment [30]. These observed novel interactions have the potential to facilitate the process of Th1-like cell recruitment and activation. Furthermore, the Th1-like cells in turn could interact with those cDCs through GAS6-AXL, IL24-NOTCH2, and IL6-IL6 receptor pairs, suggesting complex interactions between these cells within immune systems in tumors (Fig. 5h). Collectively, our iMAP may help tap further potentials of 10x in terms of exploring novel cell-cell interactions.

## Discussion

iMAP addresses the fundamental batch effect removal problem in the application of single-cell transcriptomes. It takes the gene expression profile matrices from different batches as inputs and outputs the corrected expression profiles. Our model combines the powers of both autoencoders and GANs. We deploy a novel autoencoder structure to help build disentangled batch-ignorant representations of cells. Autoencoders can retain the biological contents of cells, which is necessary for identifying the batch-specific cells, while it is difficult for them to well-mix the batch-shared cells. We further train one GAN model using extended MNN pairs between batches. These pairs, named as rwMNN pairs, searched by the representations of autoencoders and extended by a random walk-based procedure, could better encapsulate the underlying distributions of the shared cell types between batches. GANs trained on rwMNN pairs could perfectly mix the distributions of the shared cell types. On multiple benchmark datasets, iMAP shows superior performance in terms of both discerning the batch-specific cells and mixing the batch-shared cell types.

iMAP shows minimal time cost increasement when the cell number exceeds thousands, providing possible huge potentials to apply on very large-scale single-cell studies. The excellent time performance is achieved by elaborated algorithm design and powerful computational performance of GPUs (the “Methods” section). However, further improvements are still needed to decrease the time cost with respect to the number of batches. The training of iMAP, like all other deep learning-based models, involves lots of indispensable stochasticity and amounts of tunable hyperparameters implied in the network architectures and optimization procedures. It may require fine-tuning some or all of these parameters to obtain best performance on single specific application, although the default settings could already give solid performance in our tested benchmark datasets. In our current model, one dataset is specified as the anchor, whose expression profile would not be corrected. Further improvements may need to escape the selection of anchor and recover more precise single-cell transcriptomes. Although we propose a framework to interpret the working mechanism of iMAP, the two importance scores are still primarily heuristic and we expect more advancements in the interpretability of deep models for their applications on biological systems.

One main expectancy of integration of datasets from multiple sources is to fully utilize the useful sides of each of the sources. We demonstrate the application of iMAP on one study of CRC tumor-infiltrating immune cells. Cells were sequenced by two complementary scRNA-seq platforms—Smart-seq2 and 10x. iMAP could bring down the dropout-ratio of 10x to the level that is similar to that of Smart-seq2. This helped discriminate a plausible CD8^+^ T effectors cell cluster from the previous annotated ILCs. Because of the larger number of cells captured by 10x and boosted number of detected ligand-receptor genes by iMAP, we discovered novel interactions between cDCs and Th1-like T cells, such as OX40L-OX40, which may provide new insights for cancer immunotherapies. Finally, our method may be easily extended to tackle other types of single-cell measurements. We expect this work to be further improved to suit the multi-dimensional nature of the new single cell data.

## Conclusion

We present a novel unsupervised deep learning-based framework, iMAP, to address the essential batch effect removal problems in the application of scRNA-seq technologies. By comparing with nine notable batch effect removal methods and testing over 10 real-world datasets, we show that iMAP has superior and robust performance in terms of both reliably discerning the batch-specific cells and effectively integrating the batch-shared cell types. We also demonstrate the scalability of iMAP on the integration of two mouse cell atlases. The time cost increase is minimum when the cell number exceeds thousands. iMAP could discover novel cell-cell interactions between cDC subsets and T cell subsets when applied to the integration of tumor microenvironment datasets sequenced by Smart-seq2 and 10x.

## Methods

### The iMAP model

The iMAP model was initially inspired by neural style transfer methodologies from the computer vision field [17]. The essential objective of style transfer is to transfer natural images to paints plausibly created by one specific artist, while retaining the underlying image contents. Here we regarded different measuring processes of single-cell transcriptomes as specific “painting styles,” and then batch effect removal could be realized by transforming all cells into the same batch style. GAN-based models are current state-of-art frameworks for style transfer of images [39]. Although hitherto, available style transfer models are designed specialized for images and not suitable for biological datasets.

The most difficult challenge for batch effect removal is to balance the tradeoff between discerning identification of the batch-specific cell types and sufficient mixing of the batch-shared cell types. To overcome this entangled matter, we formalize our iMAP integration model into two stages, with one stage of representation learning and the other stage of batch effect removal of the original expression profiles. Specially, we elaborate a novel autoencoder structure in the first stage, to build representations of effects of biological variations disentangled from measurement noises on single-cell transcriptomes. These representations could already discriminate the batch-specific cell types and roughly mix those shared between batches. Further in the second stage, we can successfully decipher and eliminate the batch effects on the expression profile of each single cell, by virtue of the strong power of GANs for mixing cell distributions from different batches. To make GANs easily capture and match different modes of shared biological variations across batches, we only employ those cells with plausibly similar biological content in the training process to avoid the possibly detrimental mixture of the batch-specific cells and devise a specialized random walk procedure to fully cover the underlying cell type distributions. Details were further explained below.

#### Stage I: Disentangled representations of biological variations and measuring processes

We modeled the measured expression vector as the coupled effects of true biological variations and inevitable measurement noises. Although the measuring process may have distinctive effects on different cell types, it is reasonable to assume the true biological variations are independent of measuring noises. Considering that distilling the underlying biological contents from transcriptome measures is the critical step to remove the batch effects, we first designed a novel autoencoder structure to build representations of biological variations, which are expected to be disentangled from measuring noises.

Three forward neural networks are deployed in this stage, including one content encoder *E*, two generators (decoders) *G*_1_ and *G*_2_ (Fig. 1b). The inputs to the model include the expression vector of one cell denoted as ***x***, and its batch indicator vector *b*. One-hot encoding strategy is used to indicate the batch of the cell. For instance, in the case of three batches, cells from the first batch have their batch indicator vector *b* = [1, 0, 0]^*T*^. The output of the encoder *E* is denoted as *c* = *E*(*x*), which is expected to exclusively represent the biological contents of cells, and be ignorant of the measuring process. The neural network *G*_1_ is deployed to generate the representation of measurement noise *G*_1_(*b*), since the measurement noise cannot be fully captured by a simple one-hot vector. Another generator *G*_2_ is further used to finish the reconstruction of the original expression vector. The inputs to the generator *G*_2_ include both *E*(*x*) and *b*, because intuitively, it is possible for the generator to reconstruct the original measured expression vector only if both the biological content and measurement noise are simultaneously provided. The final reconstructed expression vector is *G*(*E*(*x*), *b*) = *f*(*G*_1_(*b*) + *G*_2_(*E*(*x*), *b*)), where *f* is a non-linear transformation, and is used to match the range of reconstructed vector with the original expression vector. The ReLU function *f*(*x*) = max(0, *x*) can be the default candidate for non-negative expression vectors. The reconstruction loss (*ℇ* represents expectation) can be formalized as:

$$ {L}_r={\varepsilon}_{x,b}{\left|\left|G\left(E(x),b\right)-x\right|\right|}^2 $$

The key to successfully extract biological contents of one cell is to disentangle the biological representation *c* from the corresponding cell batch indicator *b*. We achieve this by deliberately generating a random batch indicator vector $\overset{\sim }{b}$ for each cell, where randomly selected one element is set to 1 while others to 0. Well-trained generators *G*_1_ and *G*_2_, with *E*(*x*) and $\overset{\sim }{b}$ as inputs, should fabricate one cell with the same content as *x*. This inspired our content loss as:

$$ {L}_c={\varepsilon}_{x,\overset{\sim }{b}}{\left|\left|E\left(G\left(E(x),\overset{\sim }{b}\right)\right)-E(x)\right|\right|}^2 $$

In summary, the overall loss function of the first stage is:

$$ \underset{G,E}{\min }{\lambda}_c{L}_c+{\lambda}_r{L}_r $$

where *λ*_*c*_ and *λ*_*r*_ are tunable hyperparameters to make tradeoffs between the content and reconstruction loss. In our experiments, this loss function can be optimized at low operating cost, to obtain sufficiently good representations, especially for the identification of the batch-specific cells. However, the overwhelming researches in the field of deep learning have confirmed that it is hard to generate images indistinguishable from true ones by only optimizing the reconstruction loss of autoencoders [40], which inspired us to add the adversarial structures in the stage II, further removing the batch effects from the original expression profiles.

#### Stage II: Batch effect removal by GANs

Although in the ideal case, the representations built from the previous stage should be independent of the batch effects, according to our trials, it is hard to retrieve the corrected expression profiles only by the generators *G*_1_ and *G*_2_. Therefore, we further use a GAN-based model to almost perfectly match the data distributions of the shared cell types across different batches and then generate the corrected expression profiles in the stage II. The basic idea here is to transform cells from all other batches to pseudo-cells of one pre-selected “anchor” batch, and the pseudo-cells are expected to be indistinguishable from true cells of the anchor batch. By indistinguishableness, we do not pursue perfect overlap with true cells for each single pseudo-cell, but endeavor to match the distribution of pseudo-cells with the distribution of true cells with the same or similar biological contents.

We adopt a specialized MNN pair-based strategy to guide the integration, for only matching the distributions of cells from the shared cell types between two batches. An MNN pair is defined as a set of two cells from two batches respectively, that each cell is among the *k* nearest across-batch neighbors of the other cell [9]. We use the encoder output *E*(*x*) from the stage I to define MNN pairs, because these representations are supposed to be batch effect independent, resulting in a larger number of MNN pairs than using the original expression vectors, as we shown in Fig. 3e. Other methods based on MNN pairs may regard these pairs as anchors and then use a weighted averaging strategy to correct all other cells. One major potential drawback of the MNN pairs is that it is hard to assure these pairs could cover the complete distributions of cells from the shared cell types (Fig. 1d). We alternatively develop a novel random walk-based strategy to expand the MNN pair list. As shown in Fig. 1d, suppose cell *a*_1_ from batch 1 and cell *a*_2_ from batch 2 are selected as an MNN pair. Among the *k*_1_ nearest neighbors of *a*_1_ from batch 1, we randomly pick one cell *b*_1_. The same procedure would give one *b*_2_ cell from batch 2. Then, the set composed of *b*_1_ and *b*_2_ is regarded as an extended MNN pair, and also the next seed pair for random walk expansion. This process is repeated *m* times. For all MNN pairs, we could generate these kinds of new pairs. We call pairs obtained from this procedure as rwMNN pairs. The generated rwMNN pairs can better cover the distributions of matched cell types, which could facilitate the training of GANs (Fig. 3f). We argue that it is also beneficial to adopt rwMNN pairs for other MNN-based methods (Additional file 1: Fig. S11).

Next, we use those rwMNN pairs, denoted as ${\left\{{\left({x}^{(1)},{x}^{(2)}\right)}_i\right\}}_{i=1}^M$ (the superscript indexing its batch origin) to train the GAN model. This model is composed of two neural networks, one generator *G*^′^, mapping cell expression vector *x*^(1)^ to a pseudo-cell expression vector *G*^′^(*x*^(1)^), and one discriminator *D*^′^, discriminating the pseudo cell from the true expression vector *x*^(2)^. The adversarial loss is:

$$ \underset{G^{\prime }}{\min}\underset{{\mathrm{D}}^{\prime }}{\max }{\boldsymbol{\varepsilon}}_{x^{(2)}}\left[\log {D}^{\prime}\left({x}^{(2)}\right)\right]+{\boldsymbol{\varepsilon}}_{x^{(1)}}\log \left[1-{D}^{\prime}\left({G}^{\prime}\left({x}^{(1)}\right)\right)\right] $$

After training, all cells including those not in the rwMNN list could be transformed by the generator *G*^′^ to obtain the batch effect removal expression vectors.

#### Implementation details

We deploy a total of five neural networks. Compared with the network structures, the specific number of neurons for each layer is of less importance for a reasonable number of input dimensions. By default, the encoder *E* from the first stage is a *d* → 1024 → 512 → *l* three-layer (not including the input layer) network (*d* is the input dimension of expression vectors, and *l* is the dimension of content representations). The decoder *G*_1_ is a *n* → 512 → 1024 → *d* three-layer network (*n* is the number of batches), and the decoder *G*_2_ is (*n* + *l*) → 512 → 1024 → *d*. For all networks, the first two layers have a Mish non-linear activation [41], while the last layer is a linear transformation. Two parameters *λ*_*c*_ = 3, *λ*_*r*_ = 1 are used to balance the reconstruction loss and content loss. For the second stage, the generator *G*^′^ is a “shortcut connection” inspired by ResNet [42], which means *G*^′^(*x*) = *f*(*F*(*x*) + *x*) (*f* is a ReLU function), and *F* itself is an autoencoder structure, *d* → 1024 → 512 → *l* → 512 → 1024 → *d* (all layers are activated by Mish except the middle one). Be default, *l* is set to 256. The discriminator *D*^′^ is again a three-layer network *d* → 512 → 512 → 1. To facilitate and stabilize the GAN training process, adversarial losses are optimized via the WGAN-GP [43]. We adopt the Adam optimizer [44] to train the networks, with the learning rate 0.0005 for first stage and 0.0002 for the second.

In the stage II, we need to enquire the kNNs within batch and MNN pairs between batches for cells. This procedure may be compute-intensive. We randomly sample a maximum of *s* = 3000 cells from each batch to calculate all necessary pairs. Then, a locality sensitive hashing-based Python package “annoy” is adopted to quickly find the approximate nearest neighbors of each cell [45]. These make the time cost of the enquiry process is approximately constant with respect to the number of cells in each batch. The overall time cost depends only on the number of batches and network optimization parameters (such as the number of epochs for training). Hyperparameters used in this stage include *k*_1_ = *s*/100, *k* = *k*_1_/2, *m* = 50. All hyperparameters can be tunable by the user, although the default options could provide good enough results in most of our tested cases.

In order to deal with multiple datasets, we use an incremental matching manner. The sub-dataset with the largest total variance is selected as the anchor, and all other sub-datasets are processed in the decreasing order of their total variances. Every sub-dataset integrated to the anchor is appended to the anchor. Intuitively, the preferential integration order should arrange those sub-datasets with larger number of cell types firstly. If this information is available, we encourage the users to provide their own anchor and integration ordering. However, we argue that iMAP can also perform well to some extent even if the anchor sub-dataset lacks specific cell types. We demonstrate this in the “panc_rm” dataset, where the “inDrop” batch was selected as the anchor.

All jobs were run on a Linux server with 2x Intel(R) Xeon(R) CPU E5-2697 v4 @ 2.30GHz, 256G of DDR4 memory, Nvidia GTX 1080Ti GPU.

#### Explanations of gene importance

The interpretability has gradually become more and more important in the machine learning community, particularly for the applications on biological researches [46]. We adopt SHAP [26], a well-designed game theory-based method to interpret the trained neural networks of iMAP, through grading each gene to evaluate its importance for the outputs of one cell. We provide two kinds of scores to interpreting gene’s importance, each for building representations in the stage I and removing batch effects in the stage II, respectively. Specially, a three-layer neural network is connected to the output of encoder from the stage I, and trained to classify the external cell type information, while the encoder would not be trained further. Then SHAP is used to evaluate the importance of each gene for the classification outputs (Additional file 1: Fig. S1c). The other three-layer neural network is deployed to discriminate the batch-origin of each cell, and SHAP is again used to assign each gene an importance value for the classification of batch (Additional file 1: Fig. S1d). This importance value is regarded as the surrogate for evaluating the importance of one gene in removing batch effects. We expect these two importance scores could offer primary heuristics about the working mechanisms of iMAP, and the roles of genes on representing biological variations and measuring noises.

#### Preprocessing scRNA-seq datasets

Preprocessing of scRNA-seq datasets were performed under the standard Scanpy pipelines [47]. Low-quality cells were filtered if the library size or the proportion of mitochondrial gene counts was too large. The input expression vectors for iMAP were log-transformed TPM-like values. Prior to the first stage, we need to select highly variable genes for each batch to help discover the true biological variations from the noisy transcriptomes. Only those genes measured in all batches were considered. The Scanpy API “scanpy.pp.highly_variable_genes” was used to select highly variable genes in each batch respectively, although users could also use their preferred highly variable genes selection method. For the second stage, by default, we also used the selected highly variable genes. It is also possible to deal with the whole transcriptome, as we did for the “CRC” dataset. However, to make our default network structure, which is specially designed for dealing with inputs of highly variable genes (usually about two thousand), suitable to the whole transcriptome (usually about twenty thousand genes), we randomly divided the whole transcriptome into ten parts, each with about two thousand genes, and trained ten separate networks for each of them (Additional file 1: Table S5). We did not take the pre-processing steps into account to measure the time cost shown in Fig. 4d, e.

### Benchmarks

#### Datasets

Three commonly used scRNA-seq datasets were employed to evaluate the performance of different batch effect removal methods. The first dataset “panc_rm,” includes human pancreas cells measured by 5 different platforms. To measure the ability of different methods to detect the batch-specific cell types, we manually removed “ductal” cells from the “CEL-seq” dataset and “acinar,” “alpha” cells from the “inDrop” dataset. The “ductal” cell type has the largest number of cells in the “CEL-seq” sub-dataset. With their removal, the primary variance of “CEL-seq” may be determined by the second and third most numerous cell type, i.e., “acinar” and “alpha” cells. Then, we further removed these two cell types from another “inDrop” sub-dataset which was selected as the integration anchor. The second dataset “cell_lines,” is composed of three sub-datasets all sequenced by the 10x platform. Two of them are pure cell lines (“Jurkat” and “293 T”), and “Mix” is the equal mixture of “Jurkat” and “293 T.” For the “Mix” dataset, we performed the standard “Seurat” pipeline to cluster and annotate the cells. Those clusters with high expression of *XIST* were set as “293 T” while others as “Jurkat.” The third dataset “DC_rm,” consists of human DCs sequenced by the Smart-seq2 protocol. CD1C DCs in batch 1 and CD141 DCs in batch 2 were also removed, which are biologically similar.

Two recently published benchmark datasets “SCP424_PBMC” and “SCP425_cortex,” which sequenced thousands of cells from peripheral blood mononuclear cells and brain tissue respectively, with over ten protocols, covering most of single-cell and/or single-nucleus profiling methods, were also included for comparison of different methods. The log-10 K data, and meta information were downloaded from the Single Cell Portal (https://singlecell.broadinstitute.org/single_cell; Additional file 1: Table S1). We also tested the performance of iMAP on five additional datasets, with various numbers of cells, and detailed information can be found in Additional file 1: Table S1.

To test the performance, especially the time cost of iMAP for large-scale datasets, we ran iMAP on the Tabila Muris dataset, which consists of the mouse cells sequenced by two platforms, e.g., Smart-seq2 and 10x. The “UpdateSeuratObject” function updated the downloaded Seurat object to the version v3. The sequencing platforms were regarded as the batches. Another dataset containing over 600,000 cells from Human Cell Atlas was also adopted to test the scalability of iMAP, and its detailed information can be found in Additional file 1: Table S1.

The “CRC” dataset was used to test the applications of iMAP on the tumor microenvironments. Nearly 50,000 cells from human colon cancer were sequenced by either Smart-seq2 or 10x platforms. Cells from different patients sequenced by Smart-seq2 show less technical variations than those by 10x [30]. Therefore, we regarded all cells from Smart-seq2 as a single batch, and every patient sequenced by 10x was a separate batch. Cell types and tissue sources information were obtained from the original publication.

#### Benchmark methods

We compared our method with nine leading scRNA-seq batch effect removal methods: ComBat, scVI, LIGER, fastMNN, BBKNN, Harmony, Scanorama, Seurat v3, and DESC. See Additional file 1: Table S2 for detailed version information. Combat and BBKNN correction were performed using the scanpy API “scanpy.pp.combat” and “scnpy.external.pp.bbknn.” scVI was run using the default parameters and obtained latent representations were used for further analysis. The “optimizeALS” parameter of LIGER was set to “*k* = 20.” We used the “SeuratWarpper” versions of fastMNN (“RunFastMNN”) and Harmony (“RunHarmony”). Scanorama was run using the default parameter of “scanorama.correct.” The dimensions parameters of Seurat v3 were all set to “dim = 1:30.” DESC was run with the default parameters, and especially the “louvain_resolution” was set as 1.0. Because some methods cannot give the corrected expression values, we compared them by using the UMAP embeddings. All embeddings were run by using the same parameters of the Python package “umap-learn.”

#### Evaluation indices of batch effect removal

There exists an extensive list of batch effect removal evaluation indices in the literature [6]. Some widely used include kBET (k-nearest neighbor batch-effect test) [18], LISI (Local Inverse Simpson’s Index) [13], ASW (average silhouette width), and ARI (adjusted rand index). We argue that ARI and ASW are cluster-level indices and cannot reliably evaluate the mixture of cells from different batch at a local single-cell level (Additional file 1: Fig. S1a). kBET and LISI evaluate the batch mixing at a local level by comparing the batch distribution with kNNs of a cell with the global batch distribution. kBET has the advantage in evaluating the integration performance of batch-shared cell types, one drawback of which, however, is that when it measures the batch mixture, it is cell type ignorant. This may cause unfair results when the proportions of share cells types are too discrepant in different batches [13]. LISI could evaluate both the capacity of identification batch-specific cell types and the integration of batch-shared cell types, but it is hard to summarize all single cell-level LISI values into a simple statistic for comparing between various methods. kBET and LISI are nonetheless reliable metrics when appropriated employed. So, we first used these two kinds of metrics to compare different methods. For kBET, we computed the acceptance rates for each cell type separately and summarized the median value over all tested cells as the final output. For the “DC_rm” and “panc_rm” datasets, only those cell types appearing in all batches were taken into account, and since no cell type appears in all three sub-datasets of “cell_lines,” we computed the acceptance rates for the integration of “Jurkat” and “Mix” and the integration of “293 T” and “Mix,” respectively. One important parameter *k*, the number of nearest neighbors, has a large effect on the results of kBET, and following the kBET paper, a series of *k* values, which are chosen as 5%, 10%, 15%, 20%, and 25% of the total cell numbers, are adopted to run kBET. For LISI, we computed the cLISI and iLISI values for each cell, with the ideal cLISI equal to one. iLISI values of different methods are compared for each cell type separately, because the best values are cell type-specific, and determined by the number of batches having this specific cell type [13].

Considering that these indices all have their own limitations in terms of simultaneously evaluating both cell type and batch mixing, we propose two new indices to evaluate the batch mixture. Our evaluation procedure is also based on kNNs of a cell and divided into two successive steps (Additional file 1: Fig. S1b). Firstly, we classify all cells into “positive” and “negative” cells. “Positive” cells are those surrounded mostly by cells from the same cell type. Be default, one cell is assigned as “positive” only if at least 50% cells of its kNNs are with the same cell type label, otherwise “negative” (*k* is set as the minimum of 100 and the number of cells for this cell type). Then, those positive cells are further discriminated into “true” and “false” positive cells by a second dichotomous classifier. “True” positive cells are those surrounded by appropriate proportions of cells with different batches. We use the three-sigma rule of thumb to measure whether the observed batch distribution of one positive cell’s neighborhood is consistent with the global batch distribution. Considering a cell with cluster label *y*, the number of cells from cell type *y* in all *n* batches are *N*_1_, *N*_2_, ⋯, *N*_*n*_ respectively. We define *p*_*i*_ = *N*_*i*_/∑_*j*_*N*_*j*_ for *i* = 1, 2, ⋯, *n*. Then, by expectation, if we sample *k* cells from cell type *y*, the number of cells from batch *i* is equal to *kp*_*i*_. We regard a positive cell as true positive if the numbers of its neighbors from different batches are all within the range of 3 standard deviation around the expectation. This is to say, suppose kNNs of one true positive cell have the batch distribution *k*_1_, *k*_2_, ⋯, *k*_*N*_, then ${k}_i\in \left[\max \left(0,k{p}_i-3\sqrt{k{p}_i\left(1-{p}_i\right)}\right),k{p}_i+3\sqrt{k{p}_i\left(1-{p}_i\right)}\right]$ for all *i* = 1, 2, ⋯, *n*. By these two classification procedures, we could automatically identify those cells that are not mixed well. We use the proportions of positive and true positive cells as the quantitative indices to evaluate the performance of batch effect removal of different methods. This two-classifier system also provides an effective tool for visualizations of the batch effect removal results.

### Identification of significant ligand-receptor pairs

We used CellPhoneDB [48] to analyze the interactions between different cell types. One pair with *p* value less than 0.05 was regarded as statistically significant.

## Supplementary Information

**Additional file 1: Fig. S1.** Illustrations of evaluation metrics and gene importance scores. **Fig. S2.** Visualizations of iMAP batch effect removal results on the complete ‘DC’ and ‘panc’ dataset. **Fig. S3.** Visualizations of nine benchmark methods on the ‘DC_rm’ dataset. **Fig. S4.** Visualizations of nine benchmark methods on the ‘cell_lines’ dataset. **Fig. S5.** Evaluation of different methods using kBET. **Fig. S6.** Visualizations of nine benchmark methods on the ‘panc_rm’ dataset. **Fig. S7.** The performance of iMAP on the SCP424_PBMC and SCP425_cortex. **Fig. S8.** Visualizations of batch effect removal results of iMAP on four additional datasets. **Fig. S9.** Visualizations of batch effect removal results of iMAP and DESC on the ‘macaque_retina’ dataset. **Fig. S10.** iMAP’s robustness over changes of hyperparameters. **Fig. S11.** rwMNN boosts the performance of original MNN-based correction method. **Fig. S12.** Integration of large-scale datasets by iMAP. **Fig. S13.** Integration of CRC tumor-infiltrating immune cells by iMAP. **Table S1.** Detailed information of scRNA-seq datasets. **Table S2.** The versions of software used. **Table S3.** The effects of the width and the depth of networks. **Table S4.** Ablation studies of iMAP. **Table S5.** Performance of iMAP with input of all genes.

**Additional file 2.** Review history.

## Data Availability

The datasets used in this project are listed in Additional file 1: Table S1. The latest version of iMAP is freely available as a Python package on github (https://github.com/Svvord/iMAP) under the MIT license [49], and the source codes used to obtain the results presented in this article are available as a Zenodo archive with DOI 10.5281/zenodo.4461029 [50].
