# Supplementary material for: iMAP: integration of multiple single-cell datasets by adversarial paired transfer networks
Source: Genome Biol. 2021 Feb 18;22:63. doi: 10.1186/s13059-021-02280-8 (PMC7891139; doi:10.1186/s13059-021-02280-8)
Supplement: Supplementary file 1 — Additional file 1: Fig. S1. Illustrations of evaluation metrics and gene importance scores. Fig. S2. Visualizations of iMAP batch effect removal results on the complete ‘DC’ and ‘panc’ dataset. Fig. S3. Visualizations of nine benchmark methods on the ‘DC_rm’ dataset. Fig. S4. Visualizations of nine benchmark methods on the ‘cell_lines’ dataset. Fig. S5. Evaluation of different methods using kBET. Fig. S6. Visualizations of nine benchmark methods on the ‘panc_rm’ dataset. Fig. S7. The performance of iMAP on the SCP424_PBMC and SCP425_cortex. Fig. S8. Visualizations of batch effect removal results of iMAP on four additional datasets. Fig. S9. Visualizations of batch effect removal results of iMAP and DESC on the ‘macaque_retina’ dataset. Fig. S10. iMAP’s robustness over changes of hyperparameters. Fig. S11. rwMNN boosts the performance of original MNN-based correction method. Fig. S12. Integration of large-scale datasets by iMAP. Fig. S13. Integration of CRC tumor-infiltrating immune cells by iMAP. Table S1. Detailed information of scRNA-seq datasets. Table S2. The versions of software used. Table S3. The effects of the width and the depth of networks. Table S4. Ablation studies of iMAP. Table S5. Performance of iMAP with input of all genes. [file 13059_2021_2280_MOESM1_ESM.docx]

# Supplementary information

**Fig S1. Illustrations of evaluation metrics and gene importance scores. a** The drawback of cluster-level metrics. **b** Interpretations of proposed evaluation procedures. **c** Evaluation of gene importance for building representations of stage I. **d** Evaluation of gene importance for eliminating batch effects.

**Fig S2. Visualizations of iMAP batch effect removal results on the complete ‘DC’ and ‘panc’ dataset.**

**Fig S3.** **Visualizations of nine benchmark methods on the ‘DC_rm’ dataset.**

**Fig S4.** **Visualizations of nine benchmark methods on the ‘cell_lines’ dataset.**

**Fig S5. Evaluation of different methods using kBET.**

**Fig S6.** **Visualizations of nine benchmark methods on the ‘panc_rm’ dataset.**

**Fig S7. The performance of iMAP on the SCP424_PBMC and SCP425_cortex.** Visualizations of iMAP batch effect removal results on the ‘SCP424_PBMC’ (**a**) and ‘SCP425_cortex’ (**b**) datasets. Three kinds of colors are used to illustrate the cell type, batch, and evaluation information. **c** Quantitative assessments of different batch effect removal methods

**Fig S8.** **Visualizations of batch effect removal results of iMAP on four additional datasets.** Detailed information about the datasets could be found from Table S1.

**Fig S9.** **Visualizations of batch effect removal results of iMAP and DESC on the ‘macaque_retina’ dataset.** Detailed information about this dataset could be found from Table S1.

**Fig S10.** **iMAP’s robustness over changes of hyperparameters.** The effects of hyperparameters, including the number of sampling cells *s*, and the hyperparameters used for defining MNN pairs (*k*) and rwMNN pairs (*k*_1_), on the “cell_lines” (**a**) and “DC_rm” (**b**) datasets with the default settings highlighted.

**Fig S11.** **rwMNN boosts the performance of original MNN-based correction method. a** rwMNN pairs better sketching the underlying distributions than MNN pairs. **b** Visualizations of MNN and rwMNN-based batch effect removal results. **c** The number of pairs and proportion of true positive cells obtained by MNN and rwMNN pairs. **d** LISI values obtained by MNN and rwMNN-based corrections.

**Fig S12.** **Integration of large-scale datasets by iMAP. a** Tissue distributions of cells from the Smart-seq2 and 10x platform (Tabula Muris dataset). **b** Compositions of cells from the Smart-seq2 and 10x platforms within the liver tissue (Tabula Muris dataset). **c** The running time of different methods (The number of cells is 100,000). **d** UMAP plot of integration of Human Cell Atlas, including cord blood-derived cells and bone marrow-derived cells. **e** Time cost of separate processes for obtaining the results of **c**.

**Fig S13. Integration of CRC tumor-infiltrating immune cells by iMAP. a** UMAP plot colored by platforms and tissue sources. **b** UMAP plot colored by annotated cell types and specific marker genes. **c** Dropout-ratio of genes for CD8^+^ T cells and CD4^+^ T cells. **d** UMAP plot of ILCs colored by platforms, tissue sources, and original annotations.


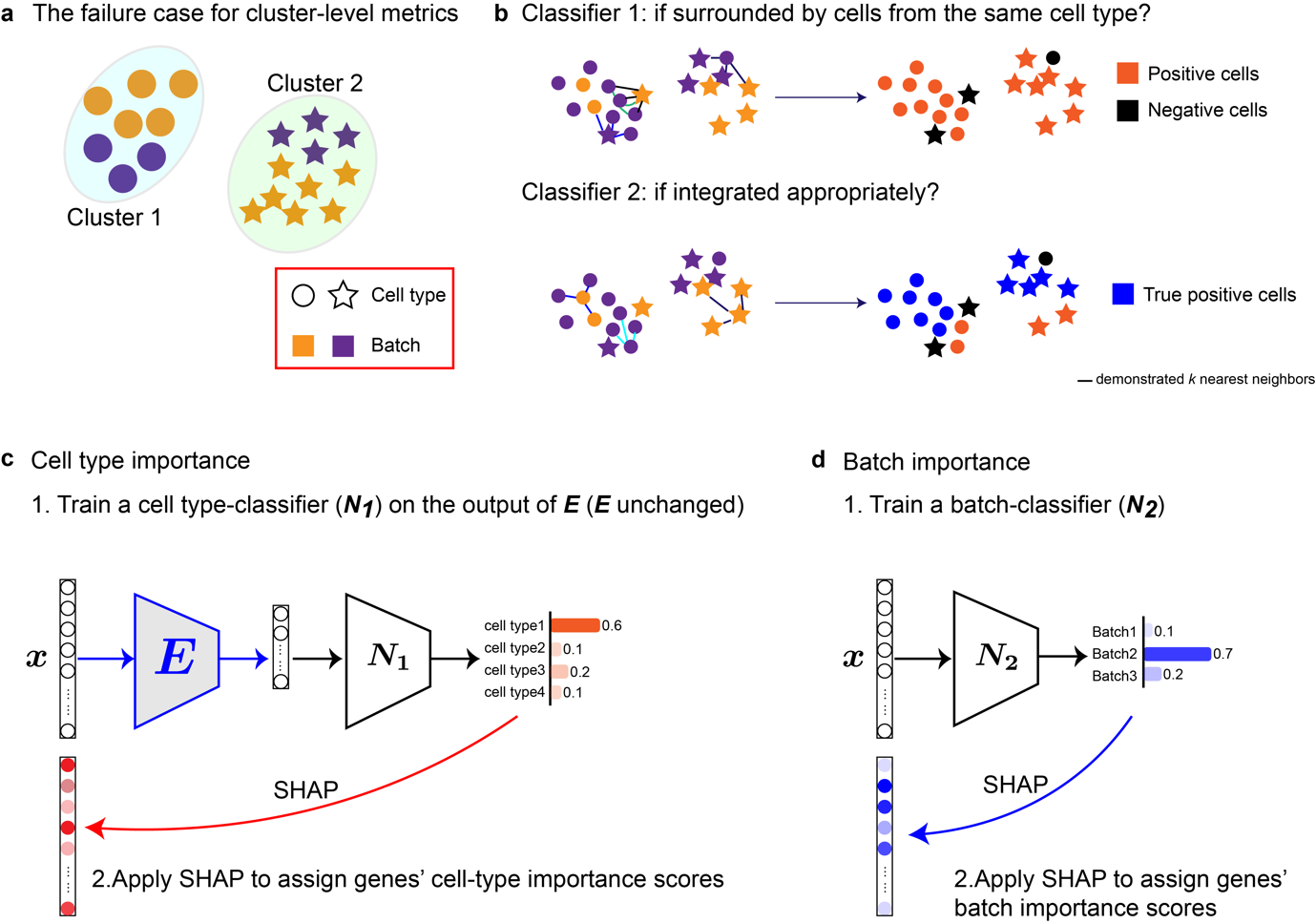


**Fig S1. Illustrations of evaluation metrics and gene importance scores. a** The drawback of cluster-level metrics. **b** Interpretations of proposed evaluation procedures. **c** Evaluation of gene importance for building representations of stage I. **d** Evaluation of gene importance for eliminating batch effects.


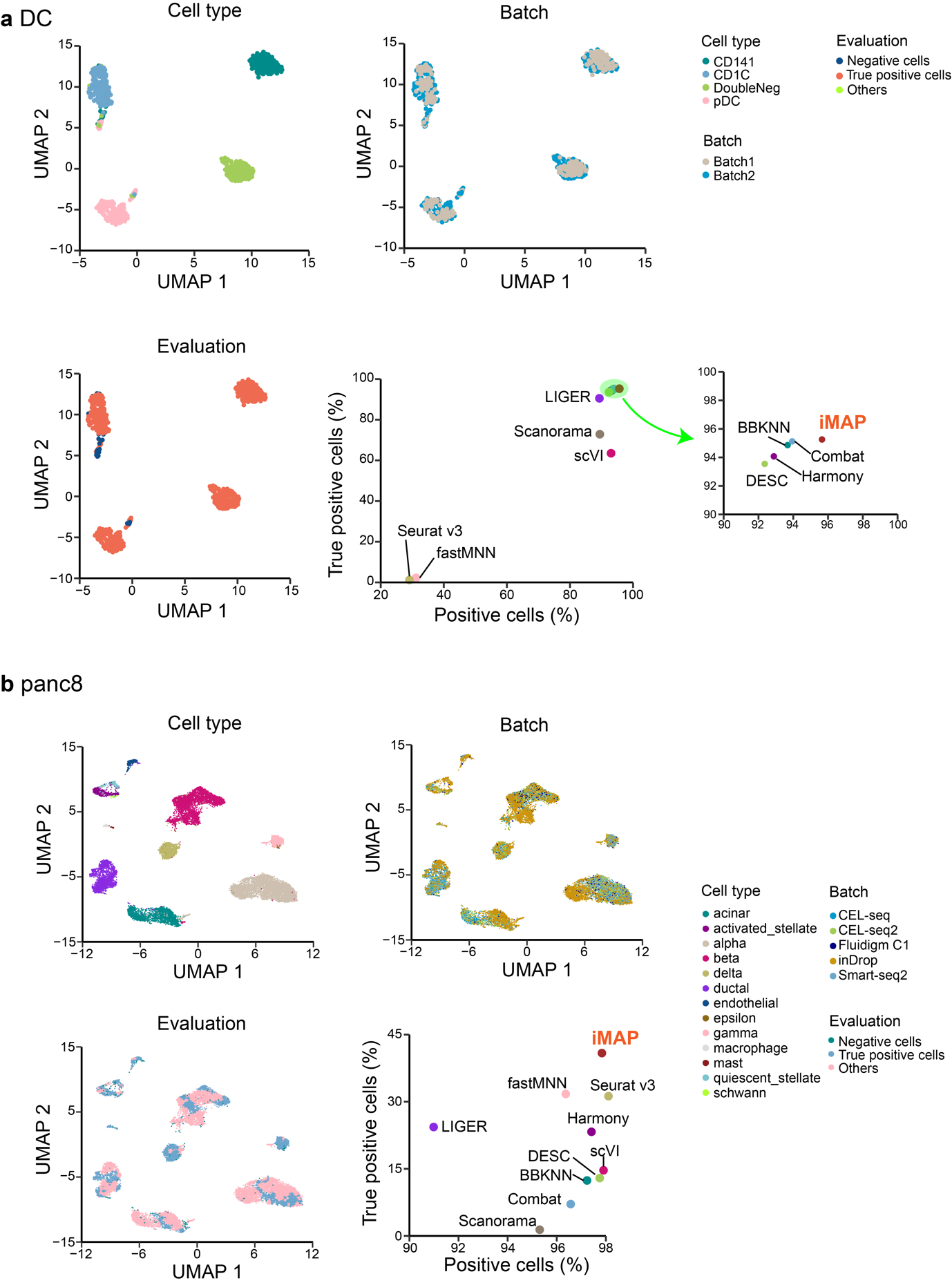


**Fig S2. Visualizations of iMAP batch effect removal results on the complete ‘DC’ and ‘panc’ dataset.**


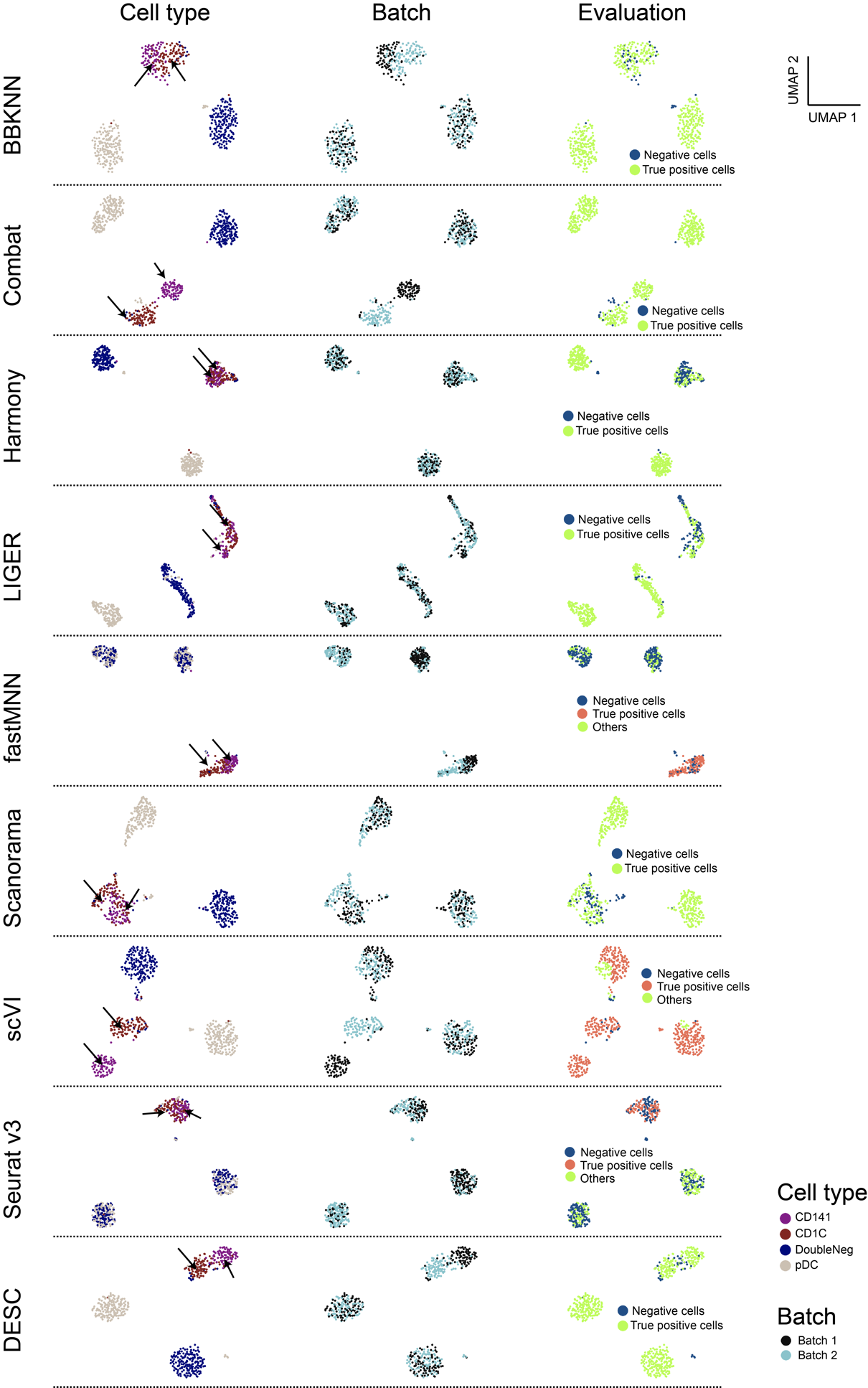


**Fig S3.** **Visualizations of nine benchmark methods on the ‘DC_rm’ dataset.**


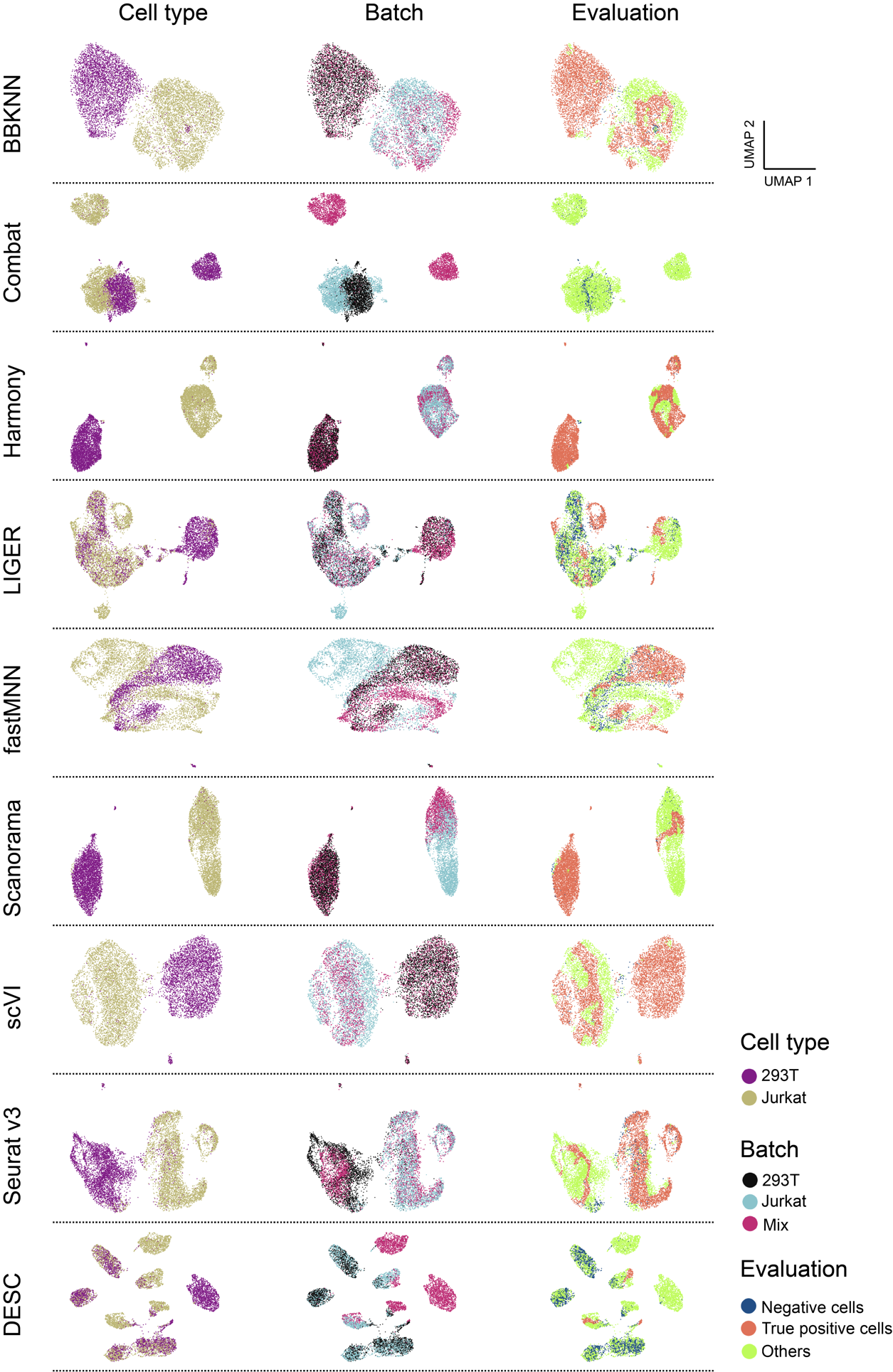


**Fig S4.** **Visualizations of nine benchmark methods on the ‘cell_lines’ dataset.**


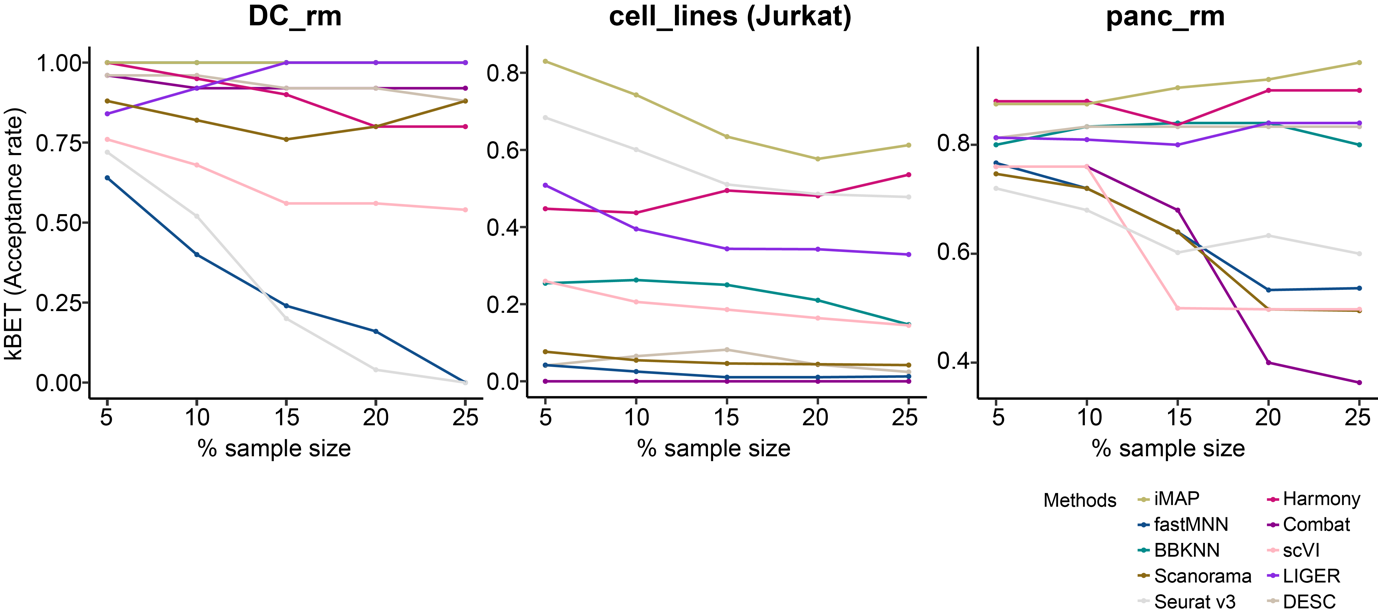


**Fig S5. Evaluation of different methods using kBET.**


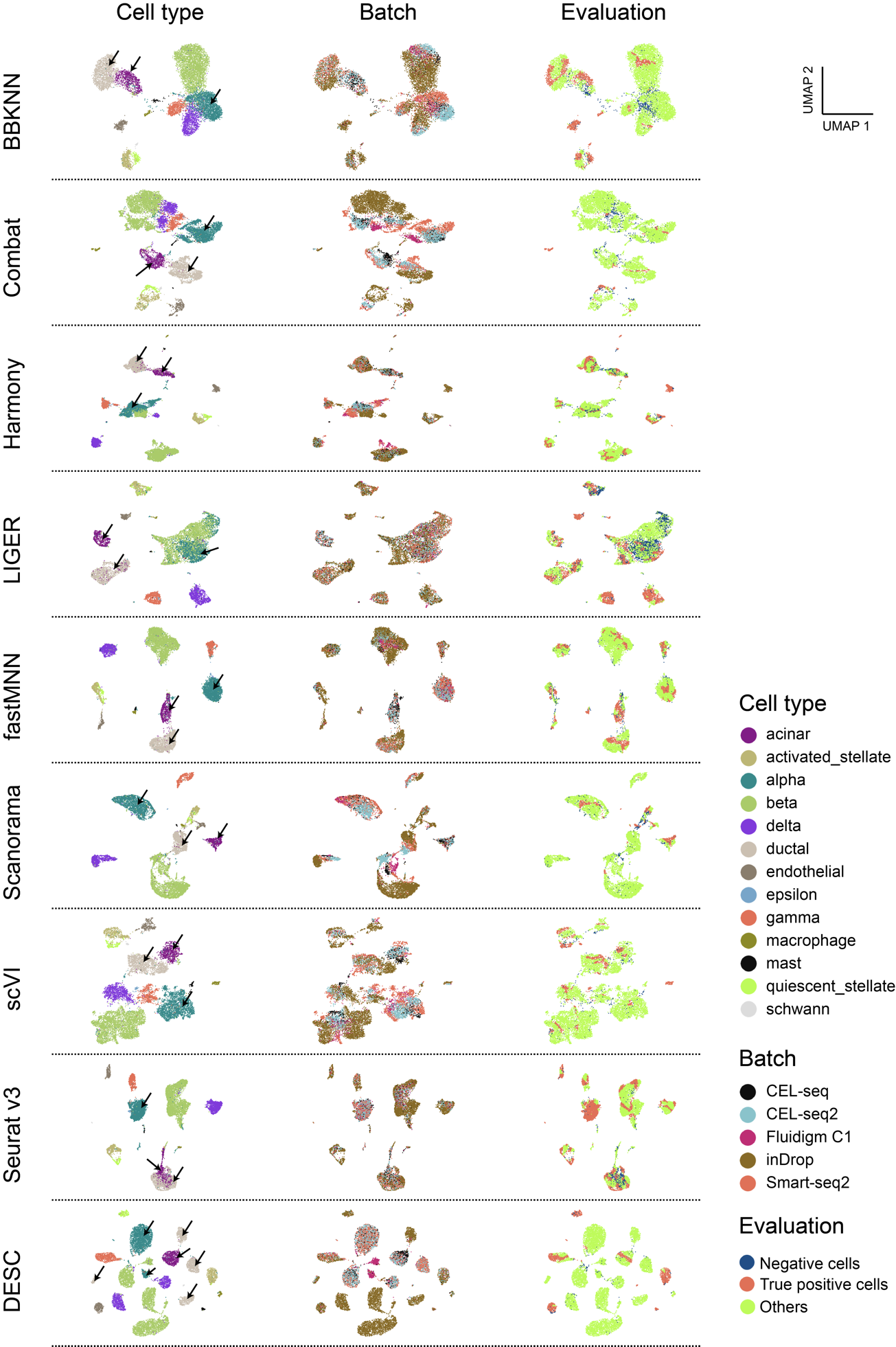


**Fig S6.** **Visualizations of nine benchmark methods on the ‘panc_rm’ dataset.**


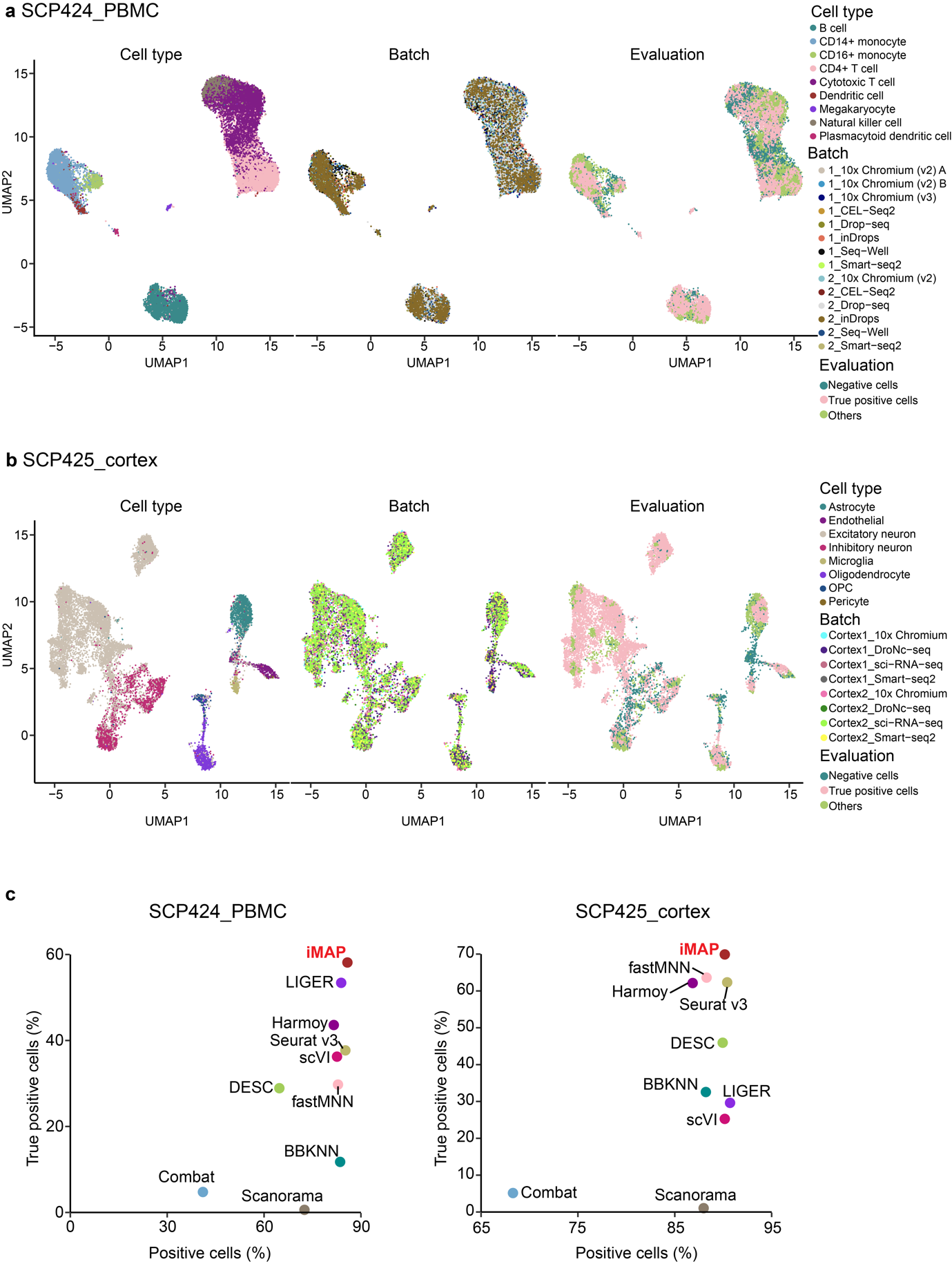


**Fig S7. The performance of iMAP on the SCP424_PBMC and SCP425_cortex.** Visualizations of iMAP batch effect removal results on the ‘SCP424_PBMC’ (**a**) and ‘SCP425_cortex’ (**b**) datasets. Three kinds of colors are used to illustrate the cell type, batch, and evaluation information. **c** Quantitative assessments of different batch effect removal methods


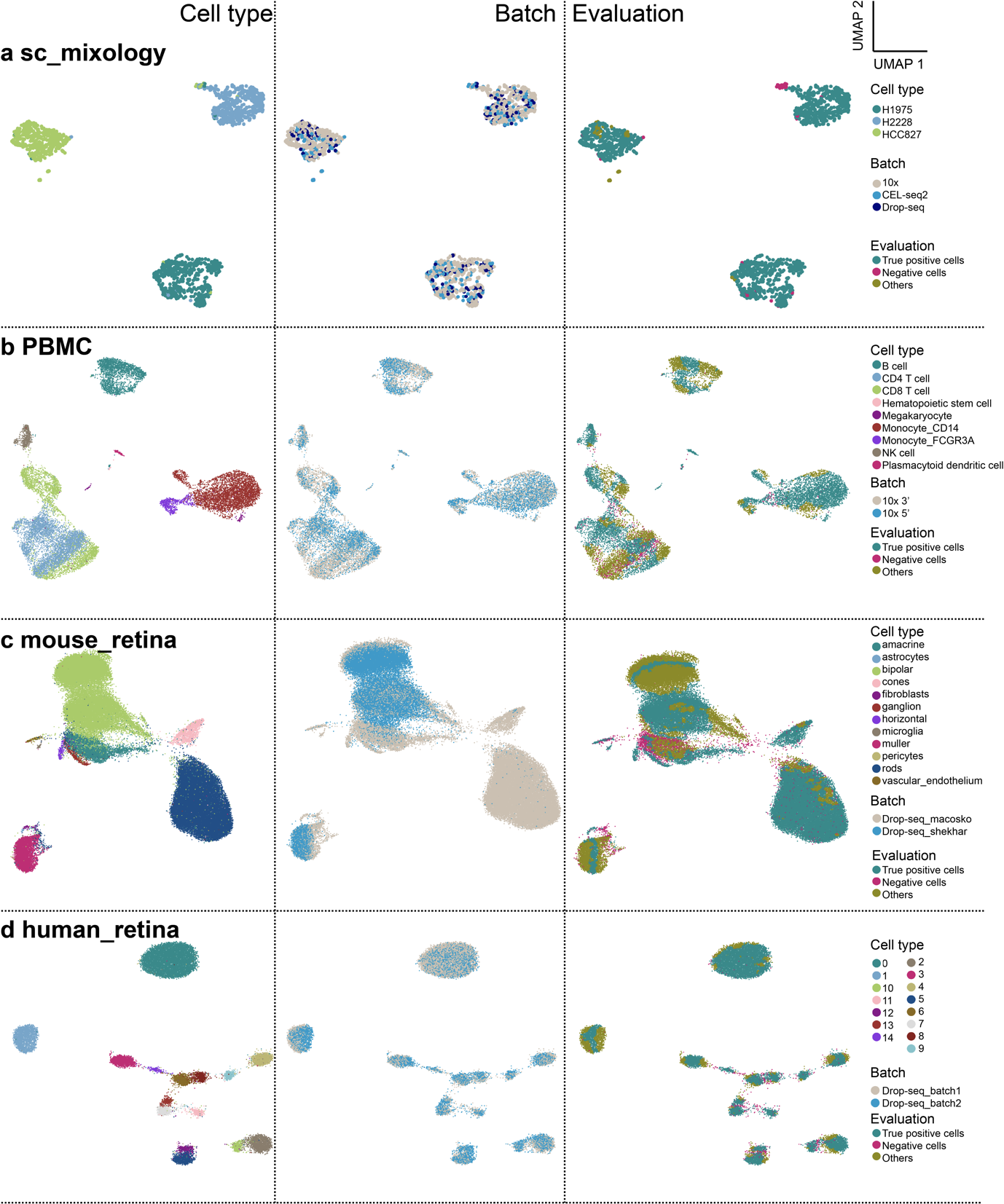


**Fig S8.** **Visualizations of batch effect removal results of iMAP on four additional datasets.** Detailed information about the datasets could be found from Table S1.


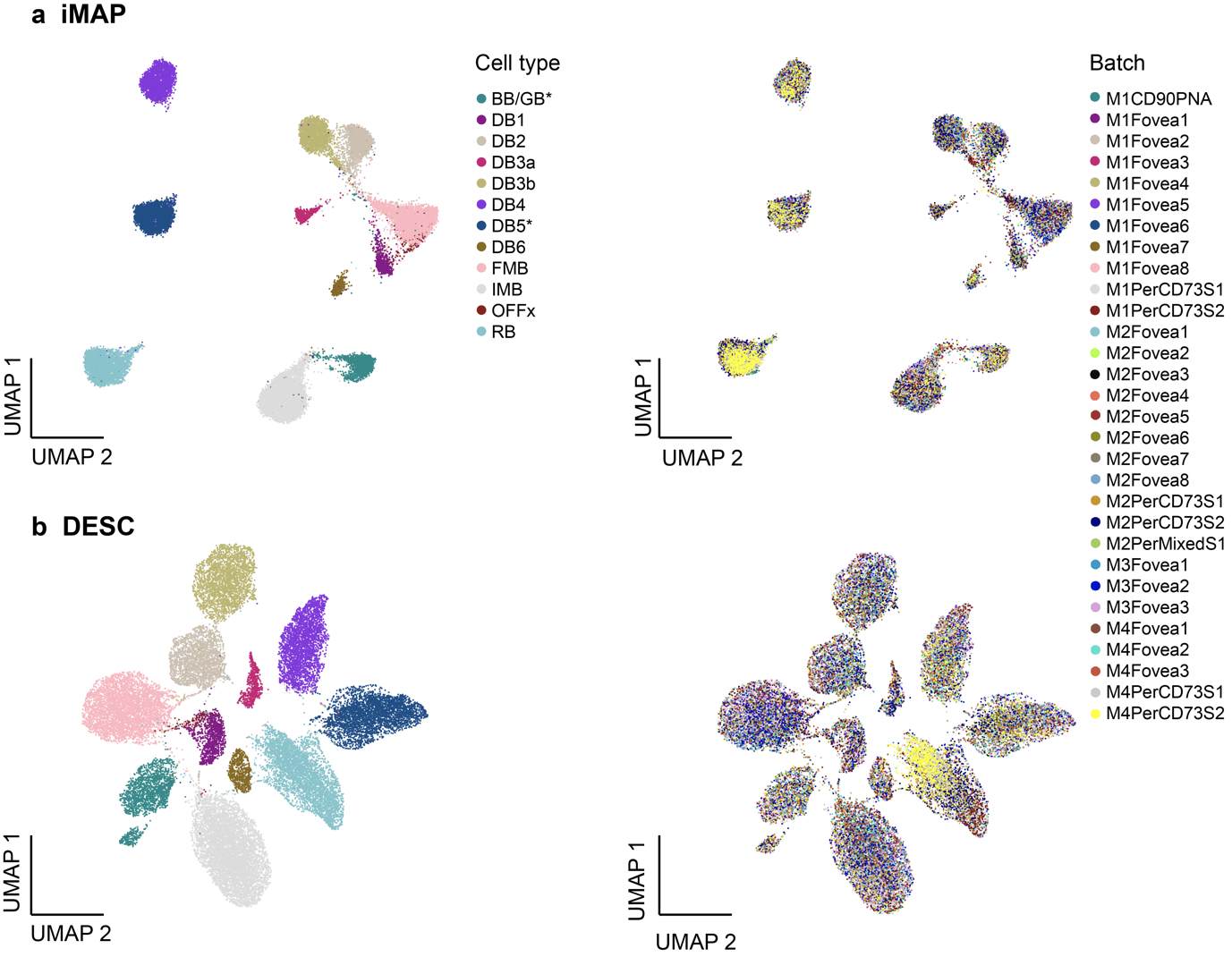


**Fig S9.** **Visualizations of batch effect removal results of iMAP and DESC on the ‘macaque_retina’ dataset.** Detailed information about this dataset could be found from Table S1.


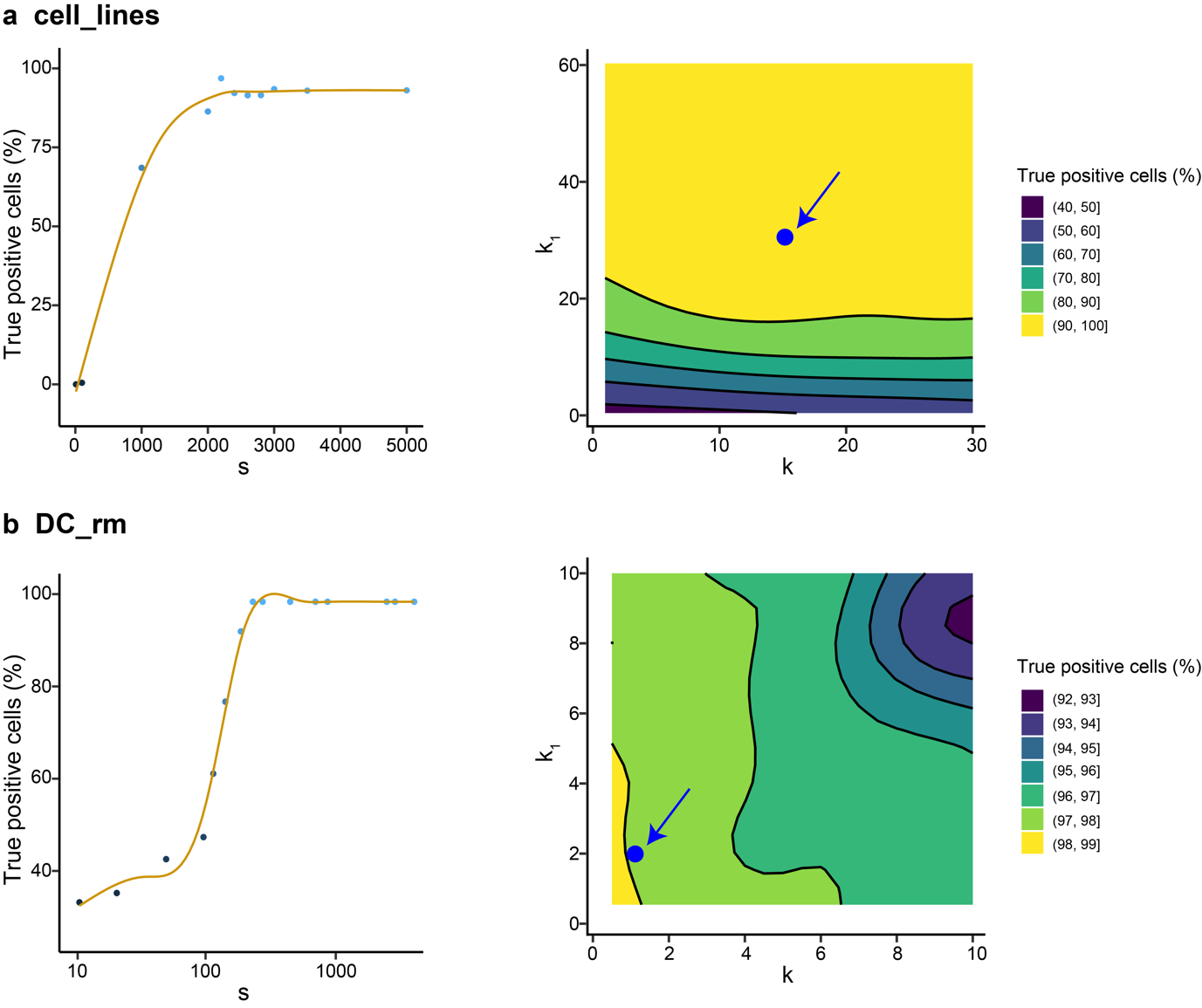


**Fig S10.** **iMAP’s robustness over changes of hyperparameters.** The effects of hyperparameters, including the number of sampling cells *s*, and the hyperparameters used for defining MNN pairs (*k*) and rwMNN pairs (*k*_1_), on the “cell_lines” (**a**) and “DC_rm” (**b**) datasets with the default settings highlighted.


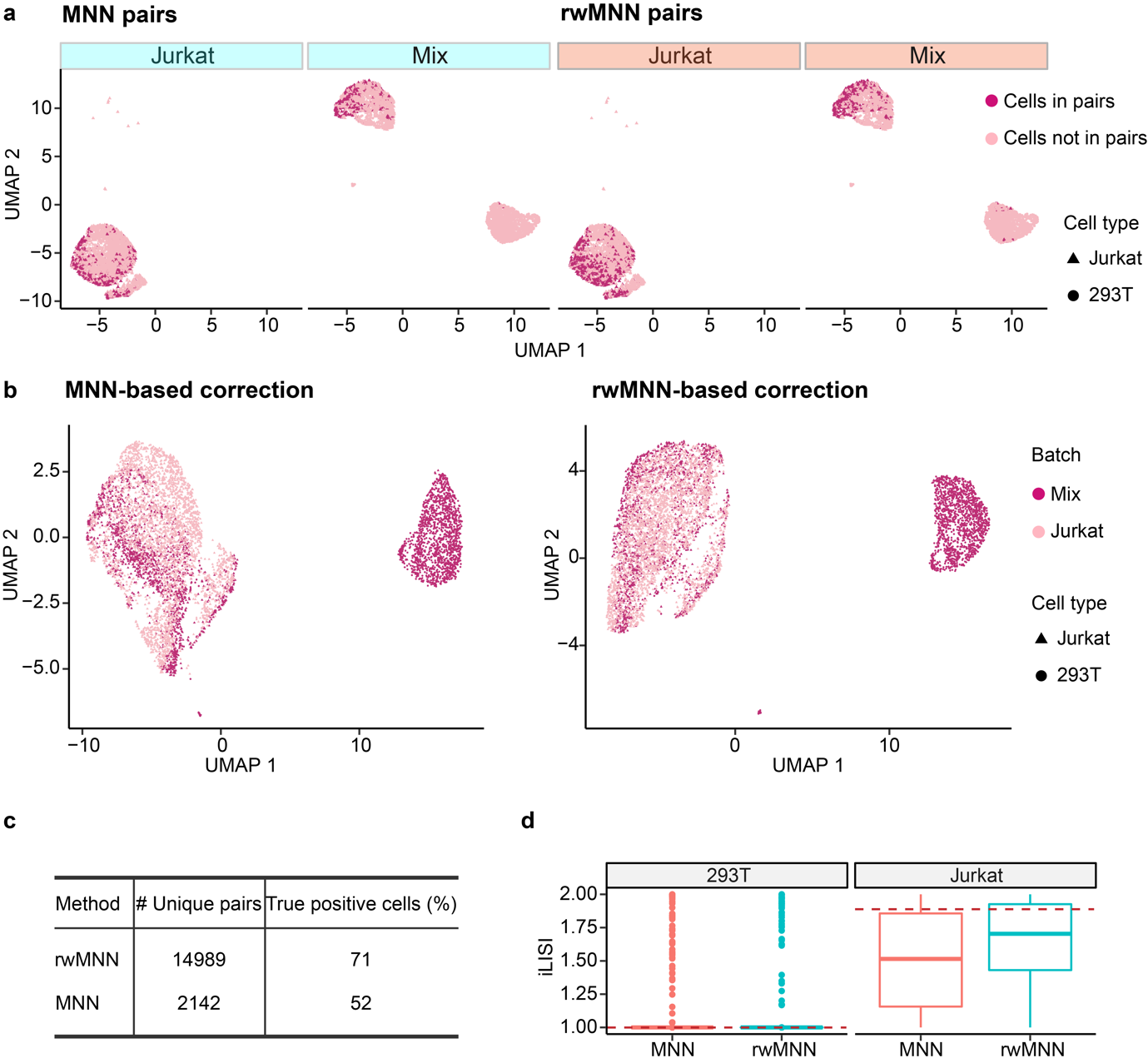


**Fig S11.** **rwMNN boosts the performance of original MNN-based correction method. a** rwMNN pairs better sketching the underlying distributions than MNN pairs. **b** Visualizations of MNN and rwMNN-based batch effect removal results. **c** The number of pairs and proportion of true positive cells obtained by MNN and rwMNN pairs. **d** LISI values obtained by MNN and rwMNN-based corrections.


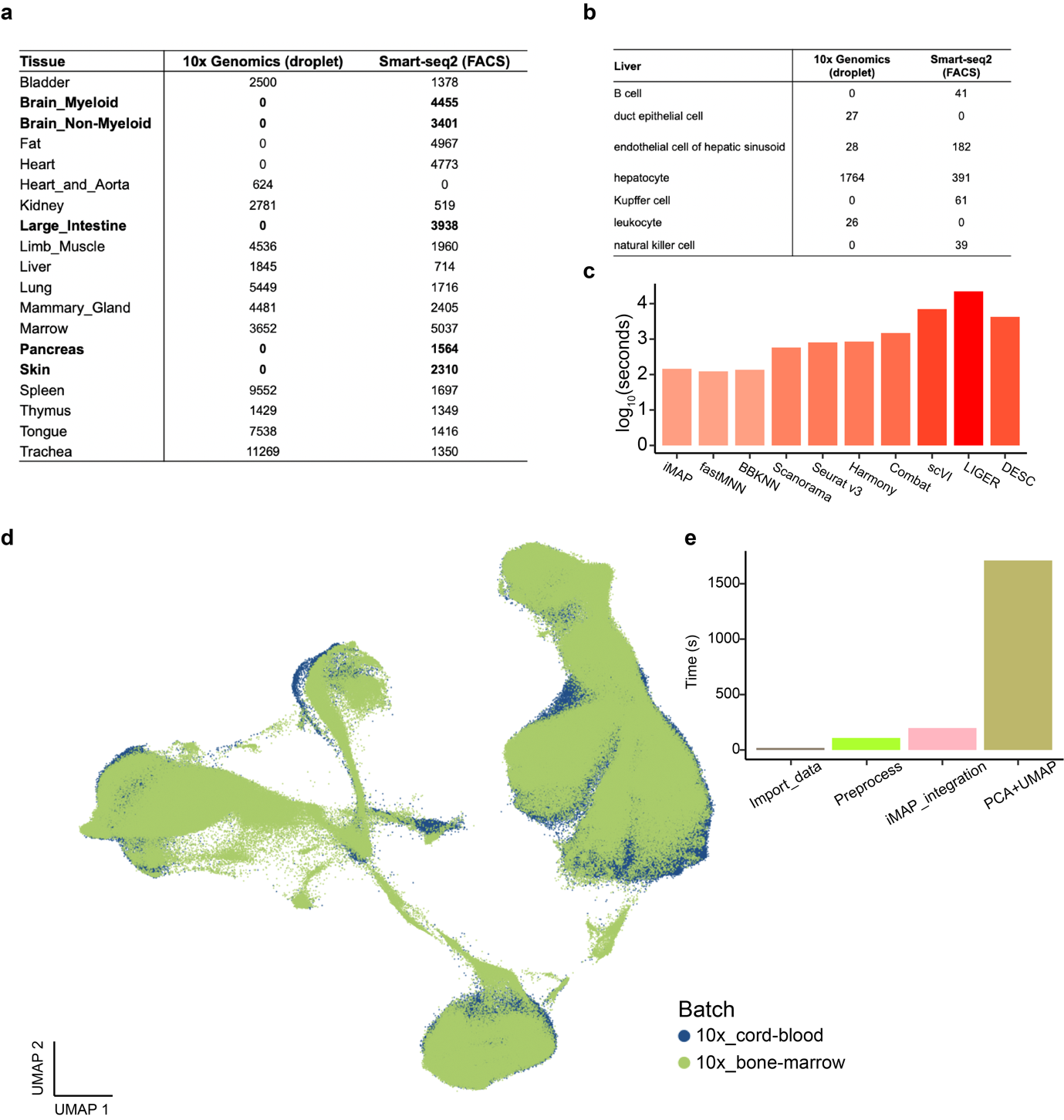


**Fig S12.** **Integration of large-scale datasets by iMAP. a** Tissue distributions of cells from the Smart-seq2 and 10x platform (Tabula Muris dataset). **b** Compositions of cells from the Smart-seq2 and 10x platforms within the liver tissue (Tabula Muris dataset). **c** The running time of different methods (The number of cells is 100,000). **d** UMAP plot of integration of Human Cell Atlas, including cord blood-derived cells and bone marrow-derived cells. **e** Time cost of separate processes for obtaining the results of **c**.


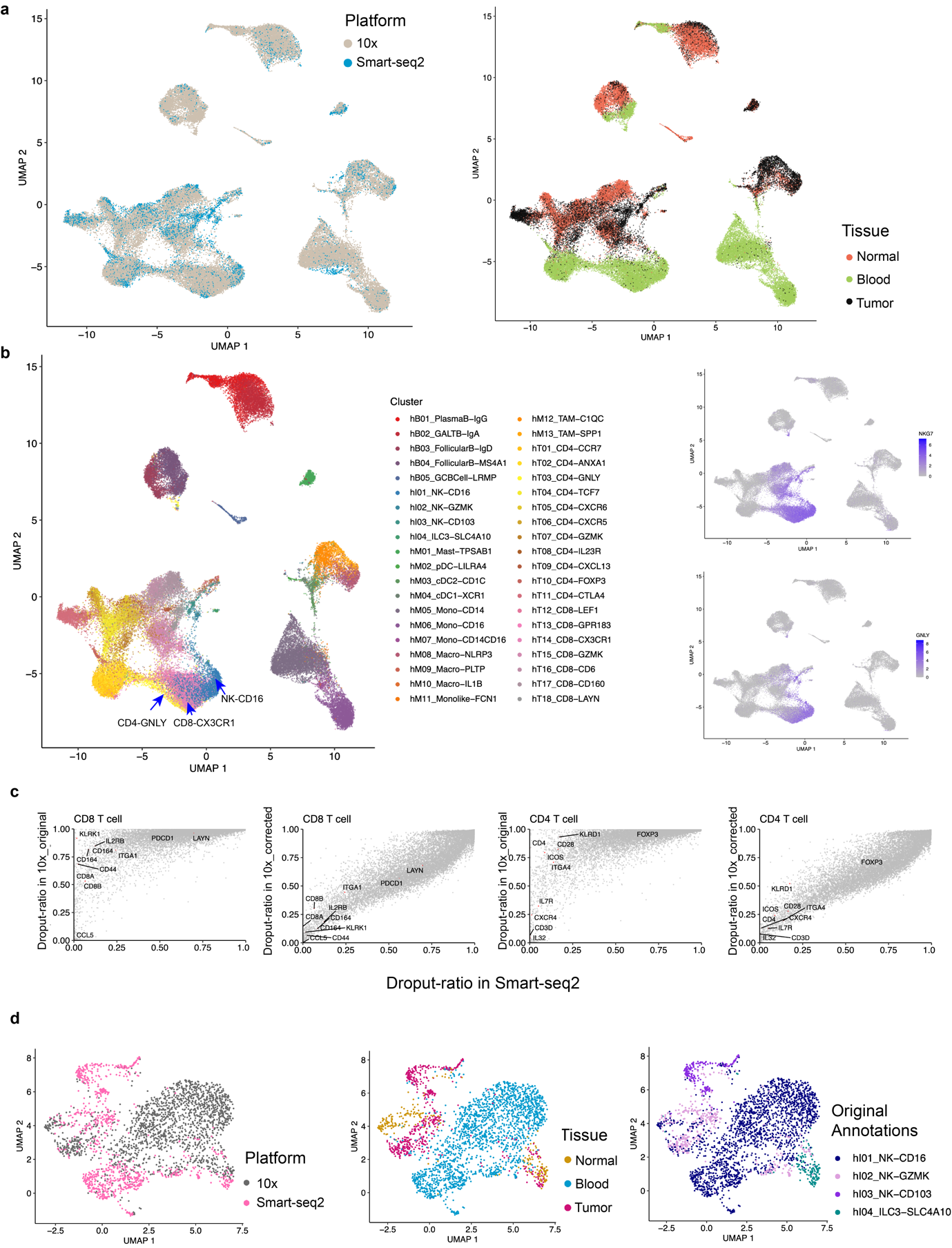


**Fig S13. Integration of CRC tumor-infiltrating immune cells by iMAP. a** UMAP plot colored by platforms and tissue sources. **b** UMAP plot colored by annotated cell types and specific marker genes. **c** Dropout-ratio of genes for CD8^+^ T cells and CD4^+^ T cells. **d** UMAP plot of ILCs colored by platforms, tissue sources, and original annotations.

### Table S1: Detailed information of scRNA-seq datasets.

| **Name** | **Source** | **Platforms** | **Cell number** |
| --- | --- | --- | --- |
| panc/panc_rm | R package: SeuratData, data(panc8) | CEL-seq | 700 |
|  |  | CEL-seq2 | 2285 |
|  |  | Fluidigm C1 | 638 |
|  |  | inDrop | 5108 |
|  |  | Smart-seq2 | 2394 |
| cell_lines | <https://support.10xgenomics.com/single-cell-gene-expression/datasets/1.1.0/jurkat> | 10x Genomics | 3053 |
|  | <https://support.10xgenomics.com/single-cell-gene-expression/datasets/1.1.0/293t> | 10x Genomics | 2676 |
|  | <https://support.10xgenomics.com/single-cell-gene-expression/datasets/1.1.0/jurkat:293t_50:50> | 10x Genomics | 3162 |
| DC_rm | <https://www.ncbi.nlm.nih.gov/geo/query/acc.cgi?acc=GSE94820> | Smart-seq2 | 286 |
|  |  | Smart-seq2 | 283 |
| SCP424_PBMC | <https://singlecell.broadinstitute.org/single_cell/study/SCP424/single-cell-comparison-pbmc-data> | 10x Chromium(V2) | 3222 |
|  |  | 10x Chromium(V2) | 3222 |
|  |  | 10x Chromium(V2) | 3362 |
|  |  | 10x Chromium(V3) | 3222 |
|  |  | CEL-seq2 | 253 |
|  |  | CEL-seq2 | 273 |
|  |  | Drop-seq | 3222 |
|  |  | Drop-seq | 3362 |
|  |  | Seq-well | 3222 |
|  |  | Seq-well | 551 |
|  |  | Smart-seq2 | 253 |
|  |  | Smart-seq2 | 273 |
|  |  | inDrop | 3222 |
|  |  | inDrop | 3362 |
| SCP425_cortex | <https://singlecell.broadinstitute.org/single_cell/study/SCP425/single-cell-comparison-cortex-data> | 10x Chromium | 1453 |
|  |  | 10x Chromium | 3944 |
|  |  | DroNc-seq | 1453 |
|  |  | DroNc-seq | 892 |
|  |  | Smart-seq2 | 295 |
|  |  | Smart-seq2 | 349 |
|  |  | Sci-RNA-seq | 1453 |
|  |  | Sci-RNA-seq | 3944 |
| Tabula_Muris | <https://figshare.com/projects/Tabula_Muris_Transcriptomic_characterization_of_20_organs_and_tissues_from_Mus_musculus_at_single_cell_resolution/27733> | Smart-seq2 | 44949 |
|  |  | 10x Genomics | 55656 |
| CRC | <https://www.ncbi.nlm.nih.gov/geo/query/acc.cgi?acc=GSE146771> | Smart-seq2 | 10468 |
|  |  | 10x Genomics | 43817 |
| sc_mixology | <https://github.com/LuyiTian/sc_mixology> | 10x Genomics | 902 |
|  |  | CEL-seq2 | 274 |
|  |  | Drop-seq | 225 |
| PBMC | <https://support.10xgenomics.com/singlecell-gene-expression/datasets/2.1.0/pbmc8k> | 10x Genomics (3’) | 8381 |
|  | <https://support.10xgenomics.com/single-cell-vdj/datasets/2.2.0/vdj_v1_hs_pbmc_5gex> | 10x Genomics (5’) | 7726 |
| human_retina | Python package: scvi.dataset.RetinaDataset | Drop-seq | 10234 |
|  |  | Drop-seq | 9595 |
| mouse_retina | <https://scrnaseq-public-datasets.s3.amazonaws.com/scater-objects/macosko.rds>  <https://scrnaseq-public-datasets.s3.amazonaws.com/scater-objects/shekhar.rds> | Drop-seq | 44808 |
|  |  | Drop-seq | 27499 |
| human_cell_atlas | <https://s3.amazonaws.com/preview-ica-expression-data/ica_cord_blood_h5.h5> | 10x Genomics | 320642 |
|  | <https://s3.amazonaws.com/preview-ica-expression-data/ica_bone_marrow_h5.h5> | 10x Genomics | 335616 |
| Macaque_retina | <https://singlecell.broadinstitute.org/single_cell/study/SCP212/molecular-specification-of-retinal-cell-types-underlying-central-and-peripheral-vision-in-primates#study-download> | 10x Genomics | 30302  (30 batches) |

### Table S2: The versions of software used.

| **Software** | **Package** | **Version** |
| --- | --- | --- |
| R |  | 4.0.2 |
|  | Seurat | 3.2.0 |
|  | SeuratData | 0.2.1 |
|  | SeuratWrappers | 0.2.0 |
|  | liger | 0.5.0 |
|  | batchelor | 1.4.0 |
|  | harmony | 1.0 |
| Python |  | 3.8.5 |
|  | umap-learn | 0.4.6 |
|  | scanpy | 1.6.0 |
|  | scanorama | 1.6 |
|  | cellphonedb | 2.1.4 |
|  | bbknn | 1.3.12 |
|  | torch | 1.1.0 |
|  | numpy | 1.18.5 |
|  | pandas | 0.23.4 |
|  | shap | 0.34.0 |
|  | annoy | 1.16.3 |
|  | scikit-learn | 0.23.2 |
|  | scvi-tools | 0.7.0 * |
|  | desc | 2.1.1 # |
| CUDA |  | release9.0(V9.0.176) |

* ran under Python 3.7.8 because of the compatibility

# ran under Python 3.6.11 because of the compatibility

### Table S3: The effects of the width and the depth of networks.

| **Dataset** | **panc_rm** | | **cell_lines** | | **DC_rm** | | **SCP425_cortex** | | **SCP424_PBMC** | |
| --- | --- | --- | --- | --- | --- | --- | --- | --- | --- | --- |
| Structure | N* | TP# | N | TP | N | TP | N | TP | N | TP |
| Original structure  $\to1024\to512\to256\to$ | 356 | 7315 | 67 | 8378 | 11 | 530 | 1343 | 9383 | 4106 | 16833 |
| $\to2048\to1024\to512\to$ | 580 | 5802 | 102 | 4877 | 11 | 484 | 2955 | 3058 | 6784 | 12245 |
| $\to512\to256\to128\to$ | 327 | 5769 | 64 | 3824 | 11 | 530 | 1307 | 7228 | 3947 | 13772 |
| $\to1024\to512\to512\to256\to$ | 379 | 7185 | 67 | 7952 | 11 | 530 | 1411 | 8618 | 4041 | 17052 |
| $\to1024\to512\to512\to512\to256\to256\to$ | 336 | 5869 | 70 | 5252 | 12 | 384 | 1392 | 7246 | 6674 | 10591 |
| $\to1024\to512\to512\to256\to128\to$ | 408 | 6099 | 68 | 5619 | 11 | 384 | 1342 | 8600 | 4034 | 14453 |
| $\to1024\to512\to256\to128\to$ | 406 | 6046 | 65 | 5428 | 13 | 500 | 1411 | 7518 | 3939 | 16194 |
| $\to1024\to256\to$ | 292 | 6569 | 64 | 8494 | 14 | 510 | 1476 | 8611 | 4198 | 14895 |
| $\to1024\to$ | 333 | 195 | 64 | 3648 | 11 | 263 | 1606 | 23 | 4314 | 3444 |

* N: the number of negative cells

# TP: the number of true positive cells

**Note:**

The default network structure of the content encoder $E$ is $d\to1024\to512\to256$, where $d$ is equal to the input dimension of expression vectors, usually about 2000, and the decoder $G_{1}$ is a $n\to512\to1024\to d$ ($n$ is the number of batches), the decoder $G_{2}$ is ($n+256)\to512\to1024\to d$, the generator $G^{'}$ is $d\to1024\to512\to256\to512\to1024\to d$. Similar as other widely used autoencoder-based structures, these networks are approximately symmetric. We modified the key component ($\to1024\to512\to256\to)$ in terms of both width and depth.

### Table S4. Ablation studies of iMAP.

| **Dataset** | **panc_rm** | | **cell_lines** | | **DC_rm** | |
| --- | --- | --- | --- | --- | --- | --- |
|  | N* | TP# | N | TP | N | TP |
| Complete iMAP | 356 | 7315 | 67 | 8378 | 11 | 530 |
| Without the stage I | 480 | 3769 | 66 | 5728 | 11 | 524 |
| Substitute the stage I with PCA | 316 | 5724 | 64 | 6841 | 17 | 453 |
| Without the content loss | 473 | 5592 | 64 | 7813 | 64 | 477 |
| Without the reconstruction loss | 2681 | 2665 | 64 | 7037 | 12 | 509 |
| Without the generator 1 | 364 | 7063 | 69 | 6919 | 12 | 529 |
| Without rwMNN | 397 | 1121 | 68 | 4626 | 19 | 208 |
| Without the stage II | 291 | 615 | 75 | 3828 | 16 | 524 |
| Substitute the stage II with MNN correction | 267 | 1314 | 63 | 5525 | 11 | 530 |

* N: the number of negative cells

# TP: the number of true positive cells

**Note: The detailed implementation of ablation studies**

1. *Without the stage I*: We deleted the whole stage I from iMAP, and directly obtained the MNN pairs from the expression vectors of highly variable genes. These pairs were employed to perform rwMNN and then stage II.
2. *Substitute the stage I with PCA*: In contrast with (1), we performed the comparison of stage I with a simple low-rank method, and obtained the MNN pairs from the space of principal components, whose dimension was set to the same as the representations of stage I (by default, 256).
3. *Without the content loss / Without the reconstruction loss*: We removed the content loss and the reconstruction loss from the stage I respectively.
4. *Without the generator 1*: We only directly concatenated the batch indicator into the input of generator 2 and removed the generator 1. The generator 2 cannot be removed from the structure, because that without it, both losses of stage I could not be computed.
5. *Without rwMNN*: We did not perform rwMNN and directly used MNN pairs obtained from the representations of stage I.
6. *Without the stage II*: We totally removed the stage II, and only used the representation from the stage I as the output.
7. *Substitute the stage II with MNN correction*: We substituted the GAN-based integration with the original MNN-based correction (still using the rwMNN pairs).

### Table S5. Performance of iMAP with input of all genes.

| **Dataset** | **All genes** | **HVGs** | **Second best performance#** |
| --- | --- | --- | --- |
| **panc_rm** | 53.5%* | 65.8% | 40.9% |
| **cell_lines** | 92.7% | 94.2% | 82.8% |
| **DC_rm** | 86.3% | 97.6% | 97.4% |

* all numbers represent the proportions of true positive cells

# The best performance obtained by other methods.
