# Supplementary material for: iMAP: integration of multiple single-cell datasets by adversarial paired transfer networks
Source: Genome Biol. 2021 Feb 18;22:63. doi: 10.1186/s13059-021-02280-8 (PMC7891139; doi:10.1186/s13059-021-02280-8)
Supplement: Supplementary file 2 — Additional file 2. Review history. [file 13059_2021_2280_MOESM2_ESM.docx]

Review History

**First round of review**

**Reviewer 1**

**Were you able to assess all statistics in the manuscript, including the appropriateness of statistical tests used?**

Yes

**Were you able to directly test the methods?**

No

**Comments to author:**

The authors resent a new method for integrating single cell datasets from multiple sources.

The method seems unnecessarily complex to me and also confusing.

Although they compare their method with other methods in the literature, the motivation of introduction of their own benchmark is perhaps not convincing.

In comparison, simpler methods like Combat and Harmony perform as good as this method on some datasets.

The usage of rwMNN pairs to train GAN needs more justification, rather than just a simple figure 3f which compares rwMNN and MNN pairs based on the classifier defined in the paper. Authors also argue in line 13 of page 12 that the rwMNN method can also be applied to other MNN-based methods, but there is no supporting data found in the paper. It will be also interesting to see comparison of methods trained on MNN pairs and not rwMNN.

The usage of 5 neural networks to over come the batch effects seems a little more complicated. A comparison with other neural network based methods other than scvI should shed more light on the performance improvements in neural network based methods.

As mentioned by the authors, the introduction of 5 neural nets introduces much stochasticity and variability, which makes it difficult to generalize the performance of the model on real world data, without much fine tuning of the hyper-parameters. The authors only discuss the number of training epochs when it comes to hyper-parameters and no others are discussed in much detail, which doesn't help while making a choice about hyper-parameters.

The benchmark datasets went through some removal of cell types, i.e "DC_rm" and "panc_rm" datasets with little justification. It will be interesting to compare the methods on the full datasets rather than the removed ones.

The introduction of the new evaluation metric based on KNNs is a good technique, but perhaps a discussion about the method in more detail, including the training details of two classifiers and their evaluation metrics can make the case more stronger on the usage of a new metric.

**Reviewer 2**

**Were you able to assess all statistics in the manuscript, including the appropriateness of statistical tests used?**

Yes: I don't have concerns with statistics in this paper.

**Were you able to directly test the methods?**

Yes

**Comments to author:**

As the scale of single-cell studies continues to grow, it becomes increasingly important to have robust and efficient methods to integrate various datasets together. For such integrative analysis, being robust to batch effect is critical. In this paper Wang et al. presented iMAP, a machine learning approach that is based on autoencoders and generative adversarial networks (GANs) to remove batch effect in single-cell RNA-seq data. In iMAP, the autoencoder is used to build low-dimensional representation and the GANs are used to remove batch effect in the original gene expression space. The use of combined autoencoders and GANs in single-cell data analysis is novel. The authors evaluated iMAP using several datasets and demonstrated its promising performance compared to other methods.

This paper is well written and easy to follow, but I have a few suggestions that I think the authors should address to make the paper stronger.

1. Although there are many methods available for batch effect correction, few can remove batch effect successfully in the original gene expression space. A recent benchmarking paper by Theis's group (https://www.biorxiv.org/content/10.1101/2020.05.22.111161v1) has also shown that batch effect correction in the gene expression space is a much harder problem. It is encouraging to see that iMAP performs better than other machine learning based methods such as scVI in batch effect correction. I have two questions about iMAP and the comparison with scVI.

a. Can iMAP also produce batch effect corrected low-dimensional representation of the data?

b. scVI can output both embedding for low-dimensional representation and batch effect corrected gene expression. When comparing with scVI, did you use embedding or the batch effect corrected gene expression?

2. For iMAP, do you need to select highly variable genes? Can you correct batch effect for all genes? For scVI, when using all genes as input, its performance tends to get worse. I am curious to know how iMAP's performance is influenced by the input genes.

3. Since iMAP needs to use MNN pairs, which can only analyze two batches at a time, I am curious to know when there are more than two batches, will the ordering of batches influence your results?

4. Evaluations of iMAP is limited to simple batch effect. I would be curious to see how iMAP performs for more complex batch effect that has multiple levels. A good testing dataset is the macaque retina data generated by Peng et al. (2019 Cell, PMCID: PMC6424338). In this dataset, there are three levels of batch effect: animal level, region level, and sample level. There are about 30 samples (batches). I will be curious to see how iMAP performs for a complex dataset like this one.

5. A recent batch effect correction method DESC (Li et al. 2020 Nature Communications; PMCID: PMC7214470) has shown better performance than scVI, Seurat 3 and MNN in removing complex batch effect in the above mentioned macaque retina data. How does iMAP compare with DESC?

6. Can you provide more details on how the importance score for each gene is calculated?

**Authors Response**

**Point-by-point responses to the reviewers’ comments:**

Point-by-point response to reviewers’ comments:

Reviewer #1: The authors present a new method for integrating single cell datasets from multiple sources. The method seems unnecessarily complex to me and also confusing. Although they compare their method with other methods in the literature, the motivation of introduction of their own benchmark is perhaps not convincing. In comparison, simpler methods like Combat and Harmony perform as good as this method on some datasets.

*We thank the reviewer for the constructive comments. Compared with traditional machine learning models, the overparameterized deep learning-based models are generally more complex and complicated, which is regarded as necessary for its robustness in different situations [1]. We apologized for not further convincing the necessity of each component of iMAP in our original version of manuscript. Here we point out the major improvements of our revised manuscript, especially after integrating all specific comments:*

*We performed the ablation studies to quantitatively measure the necessities of all components of iMAP. As shown in the new Table S3, by removing each component from the complete iMAP framework, we found that stage I is important to decrease the number of negative cells (i.e. identify batch-specific cell types), and stage II and rwMNN are critical for increasing the number of true positive cells (i.e. mix batch-shared cell types).*

*We also modified the original MNN methods [2] by the rwMNN process to demonstrate its importance, which was also not fully addresses in our original manuscript. The results were summarized in a new Supplementary Fig. S9. The details would be stated in the response to specific comments.*

*In addition, we compared iMAP with one recently published deep learning-based method (i.e. DESC [3]) to further show the performance improvements of iMAP. The results can be found in the new Fig. 2, 3, and Supplementary Fig. S2-7.*

*We added a new figure to give detailed description of our benchmark indices (Supplementary Fig. S1b). To make our benchmark results more convincing, we also applied a widely used benchmark index – LISI [4] to compare different methods, and the results also show iMAP can give consistently superior performance (See Fig. 2). However, LISI values are hard to be expressed as simple summary statistics, urging us to introduce new benchmark indices which are easy to be summarized for comparisons of different batch effect removal methods and adopted to visualize the performances.*

*For the simpler methods, we agree with the reviewer that Combat and Harmony could give adequate performance in certain datasets, but their batch effect removal may completely fail if their implicit assumptions are violated. For example, Harmony is hard to make a good tradeoff between mixture of the same cell types and identification of batch-specific cell types, especially if they are biologically similar, as we shown in the ‘DC_rm’ and ‘panc_rm’ datasets (Supplementary Fig. S3, S5). Combat can usually correctly identify batch-specific cell types, but it is hard for this kind of global modeling methods to mix all batch-shared cell types as we shown in the ‘panc_rm’ datasets (Supplementary Fig. S5). Combat also generates meaningless results in the ‘cell_lines’ dataset (Supplementary Fig. S4).*

*Basically, iMAP is designed to exploit the ability of generative adversarial networks (GANs) to match two distributions. The main difficulty here is that real biological datasets usually consist of different cell types, each of which may have possibly distinct or similar gene expression distributions. To guide GANs to mix the distributions of the same cell types from two batches, and ignore the batch-specific cell types in the stage II, we designed the stage I and the rwMNN process.*

*Stage I is necessary because we want to identify batch-specific cell types and discover more MNN pairs between the same cell types of two batches (Fig. 3e). As we discussed above, Combat-like global modeling methods have the advantage of discerning batch-specific cell types. Our stage I can be regarded as an advanced version of global modeling strategies. We input a batch indicator to help the autoencoder-based network extract batch-ignorant representations. As we demonstrate in the following figure, after stage I, batch-specific cell types, including acinar, alpha, and ductal, have already been identified, and batch-shared cell types are roughly mixed.*

*The rwMNN procedure can help extend the MNN pairs to better cover the underlying gene expression distributions in each batch. Since GANs would match the distributions from two batches, the better sketch of distributions can intuitively generate better mixture of cells. We would further discuss this in your following question.*

*In summary, we think the complexity of iMAP and each of its component is necessary for its superior and robust performance in real-world datasets. We hope these descriptions and our new analyses could clarify your confusions.*

The usage of rwMNN pairs to train GAN needs more justification, rather than just a simple figure 3f which compares rwMNN and MNN pairs based on the classifier defined in the paper. Authors also argue in line 13 of page 12 that the rwMNN method can also be applied to other MNN-based methods, but there is no supporting data found in the paper. It will be also interesting to see comparison of methods trained on MNN pairs and not rwMNN.

*We thank the reviewer for these important suggestions and comments. We appreciate the constructive suggestions to apply rwMNN pairs to other MNN-based methods, and modified the original MNN method as another demonstration of the importance of rwMNN.*

*We used two sub-datasets of ‘cell_lines’ (i.e. ‘Jurkat’ and ‘Mix’) as an example to show the effects of rwMNN in detail. As shown in the new Supplementary Fig. S9a, we first highlighted the cells selected as MNN pairs and rwMNN pairs between these two sub-datasets. rwMNN significantly increases the pairs between two datasets, with the number of unique pairs increased from 2142 to 14989 (Supplementary Fig. S9c). Of note, the rwMNN process only better sketches the underlying distributions indicated by the MNN pairs, and does not further explore those regions not captured by MNN pairs, which is important to avoid generating new false positive pairs. We then used these rwMNN pairs as seeds to perform the correction procedure of the original MNN methods [2], and as shown in Supplementary Fig. S9b, rwMNN-based correction provides better mixture of the batch-shared ‘Jurkat’ cells. Quantitative comparisons show that rwMNN boosts the proportion of true positive cells from 52% to 71%, with the iLISI values also being closer to the theoretical optimal lines (Supplementary Fig. S9d). We have now included these analyses in the updated manuscript, and thank the reviewer for these helpful suggestions, which surely help us further improve the manuscript.*

The usage of 5 neural networks to overcome the batch effects seems a little more complicated. A comparison with other neural network-based methods other than scVI should shed more light on the performance improvements in neural network-based methods.

*We appreciate this helpful suggestion. We additionally compared our method with a recently published deep learning-based method – DESC [3], which is also an autoencoder-based model, but uses a distinct integration strategy from scVI. We also noticed that scVI had made a critical update recently, and thus performed the comparison with this newest version (scvi-tools v0.7.0). We find that iMAP shows consistent better performances than scVI and DESC in all our benchmark datasets (Fig. 2 and Fig. 3b).*

*iMAP includes more networks and is obviously more complicated than those popular deep learning-based models, but we also believe that this is the key for the superior and robust performance of iMAP. To further demonstrate its robustness, we ran iMAP on over 10 real-world datasets, derived from different biological systems and sequencing technologies, and found that iMAP could consistently provide solid performances (Supplementary Fig. S6, S7).*

As mentioned by the authors, the introduction of 5 neural nets introduces much stochasticity and variability, which makes it difficult to generalize the performance of the model on real world data, without much fine tuning of the hyper-parameters. The authors only discuss the number of training epochs when it comes to hyper-parameters and no others are discussed in much detail, which doesn't help while making a choice about hyper-parameters.

*We thank the reviewer for this important comment. To further show the performance of iMAP on real-world data, we added one more complex dataset (PMCID: PMC6424338) which contains 30 batches and each with about 11 cell types in the revised manuscript, and iMAP can also accurately identify these cell types and make sufficient mixture of batch-shared cell types (Supplementary Fig. S7). We can already obtain adequate results in our all tested over 10 real world datasets with the default settings, although the fine-tuning of hyperparameters is indispensable to achieve the best performance of iMAP on one specific dataset.*

*We apologized for leaving out other hyperparameters in our original manuscript. We added analyses about other related hyperparameters, including the number of sampling cells s, and the hyperparameters used for defining MNN pairs (k) and rwMNN pairs (k1). In our default settings, s=min⁡〖{3000,#Number of cells in one batch}〗, and k and k1 are determined by s. We tested the effects of these hyperparameters on the “cell_lines” and “DC_rm” datasets by exhaustively searching a wide range of them, and find that our default settings are located in a broad region of good if not best performance. These results are illustrated in the new Supplementary Fig. S8. The most influential hyperparameters concerned with the performance of iMAP is the number of training epochs, which is the commonplace for deep learning-based models. The network structures, including the number of hidden layers and the number of nodes in each layer, are relatively robust to different datasets. This is reasonable because our input dimensions are relatively fixed due to the highly variable gene selection procedure and our training strategies for the input of the whole transcriptomes. So, we don’t suggest the users make any change to the network structures.*

*In summary, we think that our default settings of most hyperparameters are suitable for most real-world datasets, but users can further fine-tune them if they wish to experiment to achieve any improved performance.*

The benchmark datasets went through some removal of cell types, i.e "DC_rm" and "panc_rm" datasets with little justification. It will be interesting to compare the methods on the full datasets rather than the removed ones.

*We thank the reviewer for pointing out this problem. In our original version, we only compared the methods on the complete ‘panc’ datasets, and ignored the complete ‘DC’ dataset. We added the comparison on the ‘DC’ dataset in the revised Supplementary Fig. S2a. iMAP still attains the best performance on these complete datasets. We adopted the ‘DC_rm’ dataset in light of the work of Tran et al. [5] because the complete ‘DC’ dataset is easy to integrate for most batch effect removal methods. The ‘DC_rm’ dataset contains golden-standard cell type labels, and batch-specific but biologically similar cell types. In real-world applications, these kinds of biologically similar cell types may be important for novel discoveries. We introduced the ‘panc_rm’ dataset because we would like to test the performance of different methods if the biological contents of different batches are distinctive. We first removed the ‘ductal’ cells from the ‘CEL-seq’ sub-dataset, which make up the cell type with the largest number of cells in this sub-dataset. With their removal, the primary variance of ‘CEL-seq’ may be determined by the second and third most numerous cell type, i.e. ‘acinar’ and ‘alpha’ cells. Then we further removed these two cell types from another ‘inDrop’ sub-dataset that served as the anchor. We included the detailed exclusion criterion in our revised manuscript as followings:*

*“The first dataset ‘panc_rm’, includes human pancreas cells measured by 5 different platforms. To measure the ability of different methods to detect the batch-specific cell types, we manually removed ‘ductal’ cells from the ‘CEL-seq’ dataset and 'acinar', ‘alpha' cells from the ‘inDrop’ dataset. The ‘ductal’ cell type has the largest number of cells in the ‘CEL-seq’ sub-dataset. With their removal, the primary variance of ‘CEL-seq’ may be determined by the second and third most numerous cell type, i.e. ‘acinar’ and ‘alpha’ cells. Then we further removed these two cell types from another ‘inDrop’ sub-dataset which was selected as the integration anchor.”*

The introduction of the new evaluation metric based on KNNs is a good technique, but perhaps a discussion about the method in more detail, including the training details of two classifiers and their evaluation metrics can make the case much stronger on the usage of a new metric.

*We thank the reviewer for this important suggestion. We apologize for the unclear description which led to confusion, and would like to clarify that the term ‘classifier’ used here is not in a supervised learning context. To provide clear illustration, we have now included a new figure to show the details of our metrics (Supplementary Fig. S1b). The two classifiers classify each single cell based on their neighborhoods, into three classes: negative cells, true positive cells, and other positive cells. Negative cells are those surrounded by cells from other cell types. True positive cells are those positive cells whose neighborhoods are composed of appropriate numbers of cells from different batches. Given one cell type, we have its global distribution over all batches. For each cell of this cell type, its neighborhood can be regarded as one observation of the global distribution of its cell type. If the observation of local batch compositions is statistically reasonable under the global distribution, i.e. within 3 standard deviation around the expectation, we classify this cell as one true positive cell.*

*We also included the LISI values to assist assessing the performances of different methods (See Fig.2), and the reason we introduced new benchmark metrics is that LISI values are hard to be summarized as simple statistics and could not provide intuitive visualization. Compared with LISI, our benchmark metrics are easy to be summarized as simple statistics, and could facilitate the evaluation of different methods. Because each cell is classified into three classes, we can visualize the evaluation results in the UMAP plots, and intuitively observe the performance of certain methods.*

Reviewer #2: As the scale of single-cell studies continues to grow, it becomes increasingly important to have robust and efficient methods to integrate various datasets together. For such integrative analysis, being robust to batch effect is critical. In this paper Wang et al. presented iMAP, a machine learning approach that is based on autoencoders and generative adversarial networks (GANs) to remove batch effect in single-cell RNA-seq data. In iMAP, the autoencoder is used to build low-dimensional representation and the GANs are used to remove batch effect in the original gene expression space. The use of combined autoencoders and GANs in single-cell data analysis is novel. The authors evaluated iMAP using several datasets and demonstrated its promising performance compared to other methods.

*We thank the reviewer for the enthusiastic comments on the advantage and novelty of iMAP.*

This paper is well written and easy to follow, but I have a few suggestions that I think the authors should address to make the paper stronger.

1.	Although there are many methods available for batch effect correction, few can remove batch effect successfully in the original gene expression space. A recent benchmarking paper by Theis's group (https://www.biorxiv.org/content/10.1101/2020.05.22.111161v1) has also shown that batch effect correction in the gene expression space is a much harder problem. It is encouraging to see that iMAP performs better than other machine learning based methods such as scVI in batch effect correction. I have two questions about iMAP and the comparison with scVI.

a.	Can iMAP also produce batch effect corrected low-dimensional representation of the data?

b.	scVI can output both embedding for low-dimensional representation and batch effect corrected gene expression. When comparing with scVI, did you use embedding or the batch effect corrected gene expression?

*We thank the reviewer for the positive comments, especially on the ability of iMAP to remove batch effects in the original gene expression space. We regard scVI as an important method for applying machine learning, especially deep learning-based methods on the analysis of scRNA-seq datasets, and also notice that it had a great update recently. So, in our revised version, we compared iMAP with its newest version (scvi-tools v0.7.0).*

*Basically, iMAP would recover the batch effect-removed gene expression profiles, and further use the standard scRNA-seq pipeline to build low-dimensional representations of data. In the stage I, iMAP can build roughly batch effect-removed low-dimensional representations of the data. These representations can already split batch-specific cell types, but the mixture of batch-shared cell types is a little worse than those obtained further by stage II (See the following figure).*

*In theory, if we want to obtain perfect batch effect corrected low-dimensional representations of the data, the representations from stage I can be inputted to the GAN-based networks in the stage II, which may further match the distributions of representations from batch-shared cell types. However, as this reviewer pointed out, in our framework, we prefer to directly remove the batch effects in the original gene expression space, and the current structure, especially the number of units of the first hidden layer, is specially designed for the dimensions of gene expression space, which may be not suitable for the low-dimensional representations.*

*Under the standard tutorial provided by the newest scVI version (https://www.scvi-tools.org/en/0.7.0-beta.0/user_guide/notebooks/harmonization.html), we used the latent represnetations (embeddings) obtained by scVI to perform comparisons. After updated to the newest version, we observed a significant improvement of the scVI performance, as shown in the following table:*

2.	For iMAP, do you need to select highly variable genes? Can you correct batch effect for all genes? For scVI, when using all genes as input, its performance tends to get worse. I am curious to know how iMAP's performance is influenced by the input genes.

*We appreciate this important question. Highly variable genes (HVGs) selection is very important for stage I of iMAP, because this stage is designed to identify the biological variances and discern main cell types in the dataset. Currently, we adopt the default gene selection method of scanpy and isolated it as a separate step in the whole iMAP pipeline. Users can also use their preferred highly variable genes selection method, and we have now included this point in the revised manuscript.*

*The batch effect of all genes can be corrected by iMAP in stage II. In theory, the input genes are not that critical for stage II, since we can expect the GAN model match any distributions. But different numbers of input genes may require different network structures, especially the number of units in the first hidden layer. To make our current structure designed for inputs of highly variable genes, suitable to all genes from the whole transcriptomes, we adopted a novel training strategy for the whole transcriptomes, which we previously did not explain clearly in the manuscript. In details, we randomly divide the whole transcriptome into 10 parts, each with about 2 thousand genes, and train 10 separate networks for each of them. This processing strategy is also included in the Methods section of the revised manuscript as followings:*

*“For the second stage, by default, we also used the selected highly variable genes. It is also possible to deal with the whole transcriptome, as we did for the ‘CRC’ dataset. However, to make our default network structure, which is specially designed for dealing with inputs of highly variable genes (usually about two thousand), suitable to the whole transcriptome (usually about twenty thousand genes), we randomly divided the whole transcriptome into ten parts, each with about two thousand genes, and trained ten separate networks for each of them (Table S4).”*

*We also compared results by inputting HVGs with all genes, and the results can be found in Table S4. Slightly worse but acceptable results are obtained with all genes as expected, because HVGs are obviously characterized by higher signal-to-noise ratios than random gene sets.*

3.	Since iMAP needs to use MNN pairs, which can only analyze two batches at a time, I am curious to know when there are more than two batches, will the ordering of batches influence your results?

*We thank the reviewer for this question. To test the effects of the ordering of batches, we randomly sampled 20 orderings for the ‘panc_rm’ dataset, and compared the results with our heuristic strategy, which would order all datasets according to their total variances. As shown in the following figure, our heuristic strategy can yield very competitive results. And even the worst ordering performance is comparable with the second best method (Seurat v3).*

*As pointed out by the reviewer, the ordering of batches is an important potential factor that may influence the result of iMAP. We added additional descriptions in our revised manuscript to assist the user as followings:*

*“In order to deal with multiple datasets, we use an incremental matching manner. The sub-dataset with the largest total variance is selected as the anchor, and all other sub-datasets are processed in the decreasing order of their total variances. Every sub-dataset integrated to the anchor is appended to the anchor. Intuitively, the preferential integration order should arrange those sub-datasets with larger number of cell types firstly. If this information is available, we encourage the users to provide their own anchor and integration ordering.”*

4.	Evaluations of iMAP is limited to simple batch effect. I would be curious to see how iMAP performs for more complex batch effect that has multiple levels. A good testing dataset is the macaque retina data generated by Peng et al. (2019 Cell, PMCID: PMC6424338). In this dataset, there are three levels of batch effect: animal level, region level, and sample level. There are about 30 samples (batches). I will be curious to see how iMAP performs for a complex dataset like this one.

*This is an excellent point, and we thank the reviewer for this suggestion. We ran iMAP on the suggested dataset, and found that iMAP still shows great performance. We noticed this dataset was adopted as the main demonstration in the original DESC paper [3], so we also reproduced the results of DESC on this dataset, and compared the performance with iMAP. Both iMAP and DESC can give great performances on this dataset. They all correctly identify most cell types and integrate well for the batch-shared cell types. As a comparison, iMAP obtains 12278 true positive cells and DESC obtains 10384 true positive cells, indicating a little better integration of iMAP. These new results are included in the Supplementary Fig. S7 of our revised version.*

5.	A recent batch effect correction method DESC (Li et al. 2020 Nature Communications; PMCID: PMC7214470) has shown better performance than scVI, Seurat 3 and MNN in removing complex batch effect in the above mentioned macaque retina data. How does iMAP compare with DESC?

*We appreciate this valuable suggestion. As shown in the Question 4 and Supplementary Fig. S7, both DESC and iMAP could provide solid performances on the complex macaque retina dataset, indicating their superior performance in the real-world complicated datasets. We added DESC into our benchmark methods and compared its performance with iMAP and other methods in our two benchmark datasets (We noticed DESC was not optimized for the ‘cell_lines’ dataset, because two of its sub-datasets contain only one cell types. For this reason we did not include the DESC results for the ‘cell_lines’ dataset). Both DESC and iMAP perform better than scVI in the ‘DC_rm’ dataset (Fig. 2 b,c), and can correctly discern most cell types in the ‘panc_rm’ dataset, although the integration power of iMAP is slightly better than DESC (Fig. 3b).*

6.	Can you provide more details on how the importance score for each gene is calculated?

*This is an important point. In the revised version, we added a new figure to illustrate the details on how the importance scores are calculated (Supplementary Fig. S1c, d). Our procedure is based on a leading interpretable machine learning method, named SHAP. This method is developed mainly for the classification problem, and given a trained model and the output of one sample, it can assign an importance score for each input dimension showing its contribution to the model output.*

*iMAP contains two sequential stages, where stage I is important to identify accurate cell types, and stage II is mainly for eliminating the batch effects. To leverage SHAP to obtain cell type importance scores for stage I and batch importance scores for stage II respectively, we need to create a surrogate classification problem to each stage. As shown in Supplementary Fig. S1c, for obtaining cell type importance scores, it is natural to use the representations built from the encoder of stage I, as inputs to a cell-type classifier. And for the batch importance scores, we directly input the gene expression profiles to a batch-classifier (Supplementary Fig. S1d). These importance scores are only primary explorations in explaining the batch effect removal models, and may not exactly explain the genes’ importance for iMAP results, but we think they can provide reasonable heuristic information about how iMAP works.*

*Reference*

*1.	Bubeck S, Li Y, Nagaraj DJapa: A law of robustness for two-layers neural networks. 2020.*

*2.	Haghverdi L, Lun ATL, Morgan MD, Marioni JC: Batch effects in single-cell RNA-sequencing data are corrected by matching mutual nearest neighbors. Nat Biotechnology 2018, 36:421-427.*

*3.	Li X, Wang K, Lyu Y, Pan H, Zhang J, Stambolian D, Susztak K, Reilly MP, Hu G, Li M: Deep learning enables accurate clustering with batch effect removal in single-cell RNA-seq analysis. Nat Communications 2020, 11:2338.*

*4.	Korsunsky I, Millard N, Fan J, Slowikowski K, Zhang F, Wei K, Baglaenko Y, Brenner M, Loh PR, Raychaudhuri S: Fast, sensitive and accurate integration of single-cell data with Harmony. Nat Methods 2019, 16:1289-1296.*

*5.	Tran HTN, Ang KS, Chevrier M, Zhang X, Lee NYS, Goh M, Chen J: A benchmark of batch-effect correction methods for single-cell RNA sequencing data. Genome Biology 2020, 21:12.*

**Second round of review**

**Reviewer 1**

Summary

Wang et al. propose that pairing an encoder with a GAN can more effectively remove the batch effects inherent to single-cell data than existing batch-effect correction methods. They establish a new evaluation metric that overcomes limitations such as unequal distributions of cell clusters between batches. Using this metric, they compare their iMAP method to a number of popular batch integration techniques. They only apply their method to two datasets and achieve relatively small improvement over existing methods, insofar as they’ve defined performance. They claim that their method is scalable and “stable”, despite its complexity, and apply their rwMNN method to identify cell-cell interac- tions in an immune cell sub-population from a cancer dataset, purportedly to demonstrate an “interpretable” framework that few other batch-effect correction methods claim to do.

Major comments

1. The method is overall too complex and many of the components are not adequately justified. For example, the authors write that they have per- formed ablation studies but this reviewer hasn’t seen convincing evidence that such a complicated model is necessary. Rather than experiments without stage I and II (experiments which are somewhat unclear), it would be helpful to see without content or reconstruction losses, without encod- ing vs. with, with one generator but not the other, and a fair comparison to low-rank or MNN methods alone without encoding or generation. Sim- ilarly, the exploration of hyperparemeter space seems limited to only two parameters, which is inadequate given that the network depth of the en- coder and generator is shallow, which only begs the question of why the complex architecture is even necessary if complex interactions aren’t al- lowed for.

2. There remain few benchmark comparisons and a persistent inadequate use of diverse datasets. For example, the authors claim that they need a new metric to overcome kBET but do not show that their data actually suffers from imbalanced cell type proportions across batches. Furthermore, it’s hard to gauge how sensitive their method is to dataset size, sample source, type of experiment, etc. The marginal improvement of iMAP over other methods seems to be too small to reach significance but it is hard to gauge how iMAP realistically compares to other data, given there are really only two trials. Can the authors simulate benchmark comparison data using existing single-cell simulations (such as splatter or symsim?) to more systematically explore their method, its value relative to other methods, and its sensitivities to important single-cell parameters such as size and noise?

3. The descriptions of iMAP’s stability and interpretability are mis-leading. The authors do not really make it clear what they mean by stability, i.e., wstability with respect to what and relative to what? Just because training (or overfitting) plateas after X epochs hardly means that the ar- chitecture is inherently stable. In fact, architectures involving adversarial networks are prone to mode collapse and infamously unstable. Further- more, GANs are not broadly considered interpretable models. This “re- sults” section calls into question the whole paper for me. In particular, training stability should not serve as a proxy for robustness that matters to scRNA-seq researchers (stability in light of biological or experimental variability). Furthermore, highlighting one gene without clearly describing how they performed feature-wise attribution does not convince me that their method is “interpretable”. o

Minor comments

1. The pre-processing of the human pancreas datasets seems likely to largely influence performance. I suggest doing a sensitivity analysis here where cell types are not removed from the dataset. Does iMAP still perform better than other methods in these more realistic scenarios? The results	are hard to follow here so perhaps the authors did this but could clarify	this section.

2. The section on large-scale datasets is not a fair comparison, necessar- ily, as methods such as BB-kNN show scalability of integration across orders of magnitude, rather than just 100,000 cells. A more fair com- parison would be a discussion as to how iMAP has unique advantage on small/medium/large datasets but this is impossible to gauge with the current discussion because even though they downsample, they do not downsample and compare their method to others, which seems to be the key piece of insight of interest which is missing

3. There is poor justification for throwing together an autoencoder with a GAN that could be better explored. Interpolation in an autoencoder’s latent space is typically meaningless, so it is unclear why it is necessary to use an autoencoder for preseving biological content, when an appropriately trained or constrained GAN can (and has for single-cell data) generate realistic single-cell data, conditional on some label. Can the authors more systematically explore hyperparameters and ablation studies with various autoencoder and GAN architectures? Or, for example, how do low rank approximations compare to the encoder representation?

4. Nhu Tran et al. 2020 propose five evaluation metrics to compare batch-effect correction methods. Why not do the same and use a similar dataset to frame iMAP?

**Reviewer 2**

The authors have done a great job addressing my previous concerns. I don't have any additional comments.

**Authors Response**

**Point-by-point responses to the reviewers’ comments:**

Point-by-point response to reviewers’ comments:

Reviewer #1: Summary

Wang et al. propose that pairing an encoder with a GAN can more effectively remove the batch effects inherent to single-cell data than existing batch-effect correction methods. They establish a new evaluation metric that overcomes limitations such as unequal distributions of cell clusters between batches. Using this metric, they compare their iMAP method to a number of popular batch integration techniques. They only apply their method to two datasets and achieve relatively small improvement over existing methods, insofar as they’ve defined performance. They claim that their method is scalable and “stable”, despite its complexity, and apply their rwMNN method to identify cell-cell interactions in an immune cell sub-population from a cancer dataset, purportedly to demonstrate an “interpretable” framework that few other batch-effect correction methods claim to do.

*We appreciate the suggestive comments from the reviewer, and these comments give us great opportunities to improve the manuscript. Here we provide a summary of our major revisions:*

*We have conducted ablation studies to show the necessity of each component of iMAP, including removing certain stages or network structures, substituting some parts of network structures with simple low-rank approximation methods or basic MNN-based corrections.*

*For the concerns about hyperparameters, we have now additionally analyzed the effects of the width and depth of the networks, to guide the application of iMAP on real-world datasets.*

*One additional commonly used metric, i.e. kBET, has now been enrolled in to compare iMAP with nine batch effect removal methods, further substantiating the superior performance of iMAP.*

*We have leveraged two more recently published benchmark datasets, which sequenced thousands of cells from different sources with over ten protocols, to make the benchmark more comprehensive.*

*We have performed the time cost comparisons of iMAP with other methods and further demonstrated the scalability of iMAP.*

*Detailed response can be found under each comment, and we hope the updated manuscript can clarify the concerns of the reviewer.*

Major comments

1. The method is overall too complex and many of the components are not adequately justified. For example, the authors write that they have performed ablation studies but this reviewer hasn’t seen convincing evidence that such a complicated model is necessary. Rather than experiments without stage I and II (experiments which are somewhat unclear), it would be helpful to see without content or reconstruction losses, without encoding vs. with, with one generator but not the other, and a fair comparison to low-rank or MNN methods alone without encoding or generation. Similarly, the exploration of hyperparameter space seems limited to only two parameters, which is inadequate given that the network depth of the encoder and generator is shallow, which only begs the question of why the complex architecture is even necessary if complex interactions aren’t allowed for.

*We thank the reviewer for these comments. We have now systematically performed additional experiments for further convincing the necessity of each component of iMAP. As shown in the new Table S4, current ablation studies include:*

*Without the stage I: We deleted the whole stage I from iMAP, and directly obtained the MNN pairs from the expression vectors of highly variable genes. These pairs were employed to perform rwMNN and then stage II. The performance was dramatically reduced in the ‘cell_lines’ and ‘panc_rm’ datasets.*

*Substitute the stage I with PCA: In contrast with (1), we performed the comparison of stage I with a simple low-rank method, and obtained the MNN pairs from the space of principal components, whose dimension was set to the same as the representations of stage I (by default, 256). The performance was better than (1) in the ‘cell_lines’ and ‘panc_rm’ datasets, but still much worse than the complete iMAP. For the ‘DC_rm’ dataset with a much lower number of cells than the above two datasets, PCA could even give worse results than (1).*

*Without the content loss / Without the reconstruction loss: The detailed components of stage I were then investigated as suggested. We recognize that the content loss and the reconstruction loss are complementary, and the optimization of one of them would benefit the other, since they both pursue the accurate reconstructed expression profiles (Figure 1c). However, the content loss is important for the representations to be ignorant of the batch, which cannot be attained by the reconstruction loss, and without the reconstruction loss, there exist trivial representations which generate reconstructed vectors deviating from the original expression profiles and cannot preserve the biological contents. The performance of iMAP was drastically destroyed in the ‘panc_rm’ dataset after removing the reconstruction loss, and the number of negative cells was significantly increased in the ‘DC_rm’ dataset without the content loss. In all three datasets, removing either the content loss or the reconstruction loss incurred reduced performance.*

*Without the generator 1: As shown in Figure 1b, the reconstructed expression profile is generated as the sum of outputs from two generators, where generator 1 accepts the batch indicator as its input. One alternative scenario is that we could only directly concatenate the batch indicator into the input of generator 2 and remove the generator 1. This structure could also give sensible results on all three benchmark datasets, which were inferior to those obtained from our original structure, however. The generator 2 cannot be removed from the structure, because that, without it, both losses of stage I could not be computed.*

*Without rwMNN: As we depicted in the Supplementary Fig. S11, rwMNN is of vital importance to help the MNN pairs cover the underlying distributions of shared cell types between different batches. If we did not perform rwMNN and directly used MNN pairs obtained from the representations of stage I, the integration performance of iMAP was deteriorated in all three benchmark datasets.*

*Without the stage II: During the stage II, GAN-based network is leveraged to match the distribution of shared cell types between different batches. We first totally removed the stage II, and only used the representation from the stage I as the output. As shown in the Table S4, the performance was reduced on the ‘panc_rm’ and ‘cell_lines’ datasets.*

*Substitute the stage II with MNN correction: Then we substituted the GAN-based integration with the original MNN-based correction [1], and found that the performance was elevated compared to that of (6), but was still much worse than the complete iMAP framework.*

*Next, we analyzed the effect of network structure, including the width and depth, on the performance of iMAP. The default network structure of the content encoder E is d→1024→512→256, where d is equal to the input dimension of expression vectors, usually about 2000, and the generator G_1 is n→512→1024→d (n is the number of batches), the generator G_2 is (n+256)→512→1024→d, the generator G^' is d→1024→512→256→512→1024→d. Similar to other widely used autoencoder-based structures, these networks are approximately symmetric. We modified the key component (→1024→512→256→) in terms of both width and depth to investigate the effects of network structure. As shown in the new Table S3, modest adjustments of the depth of the network have subtle impacts on the final performance, while the change of the width can significantly reduce the performance on the ‘panc_rm’ and ‘cell_lines’ datasets. These results are reasonable and generalizable across different datasets considering our specialized training strategy that the input dimension is relatively stationary and during each epoch, only a fixed number of cells are employed. Taking together with our previous analyses of other hyperparameters, including the number of training epochs (Figure 3c), the number of sampling cells s, and the hyperparameters used for defining MNN pairs (k) and rwMNN pairs (k1) (Supplementary Fig. S11), we suggest that fine-tuning should be focused on k and k1 to define pairs, and the number of training epochs, although the current default settings are suitable for most real-world datasets.*

*In summary, we think that the current training strategies, network structures, hyperparameter settings, combined with the two-stage framework of iMAP, cooperatively grant the superior and robust performance on our tested over 10 real-world datasets. These results are included in the revised manuscript, and we thank the reviewer for these concerns, which helped us further improve the manuscript.*

2. There remain few benchmark comparisons and a persistent inadequate use of diverse datasets. For example, the authors claim that they need a new metric to overcome kBET but do not show that their data actually suffers from imbalanced cell type proportions across batches. Furthermore, it’s hard to gauge how sensitive their method is to dataset size, sample source, type of experiment, etc. The marginal improvement of iMAP over other methods seems to be too small to reach significance but it is hard to gauge how iMAP realistically compares to other data, given there are really only two trials. Can the authors simulate benchmark comparison data using existing single-cell simulations (such as splatter or symsim?) to more systematically explore their method, its value relative to other methods, and its sensitivities to important single-cell parameters such as size and noise?

*We thank the reviewer for this question. We did not apply kBET to compare different methods because kBET is not designed to measure the capacity of identification of batch-specific cell types, which can be regarded as an extreme case of imbalanced cell type proportions. The primary concern about the balance between the identification of batch-specific cell types and the integration of batch-shared cell types drives us to adopt another widely used LISI metrics and propose novel metrics, and we leveraged the original three benchmark datasets because they all contain batch-specific cell types, some of which are even biologically similar. Nonetheless, we admit the advantage of kBET in evaluating the integration performance of batch-shared cell types, and additionally used kBET to compare different methods confined to these batch-shared cell types. The detailed operation is adapted from kBET’s online tutorial, and included in the revised manuscript as followings:*

*“For kBET, we computed the acceptance rates for each cell type separately, and summarized the median value over all tested cells as the final output. For the ‘DC_rm’ and ‘panc_rm’ datasets, only those cell types appearing in all batches were taken into account, and since no cell type appears in all three sub-datasets of ‘cell_lines’, we computed the acceptance rates for the integration of ‘Jurkat’ and ‘Mix’ and the integration of ‘293T’ and ‘Mix’, respectively. One important parameter k, the number of nearest neighbors, has a large effect on the results of kBET, and following the kBET paper, a series of k values, which are chosen as 5%, 10%, 15%, 20%, and 25% of the total cell numbers, are adopted to run kBET.”*

*The results of kBET on our three benchmark datasets can be found in the new Supplementary Fig. S5, and iMAP can still achieve the best performances.*

*We appreciate the concern by the reviewer about the number and diversity of benchmark datasets. In the original manuscript, we elaborately selected three datasets to decipher the relative weakness of other methods. For example, Combat provided comparable performance over iMAP in the ‘DC_rm’ dataset, but it notably failed in the ‘cell_lines’ dataset, while as Harmony made great integration for the ‘cell_lines’ dataset, it could not reliably identify the biologically similar cell type in the ‘DC_rm’ dataset (Supplementary Fig. S3, S4). And in the ‘panc_rm’ dataset, iMAP improved over all other methods by a wide margin (Figure 3b). In contrast to simulation-based datasets, we believe that real-world datasets are much more important to be compared between different methods. However, we admit the original benchmark datasets are limited in terms of the number of cells, the diversity of sequencing protocols, and the types of sample sources. We then additionally used iMAP and all other nine batch effect removal methods on two recently published benchmark datasets [2], which sequenced thousands of cells from peripheral blood mononuclear cells and brain tissue respectively, with over ten protocols, covering most of single-cell and/or single-nucleus profiling methods (Table S1). As shown in the new Supplementary Fig. S7, only iMAP could provide consistently solid performances on these two complicated datasets. Altogether, we ran iMAP on over 10 real-world datasets (Table S1; Supplementary S8, S9), derived from different biological systems and sequencing technologies, and although we did not compare with other methods for all of these datasets, these results demonstrate the robustness of iMAP on real-world datasets.*

*In summary, by adding kBET and new benchmark datasets, we further show the superior performance of iMAP over other methods, and we hope these results can clarify your concerns.*

3. The descriptions of iMAP’s stability and interpretability are mis-leading. The authors do not really make it clear what they mean by stability, i.e., stability with respect to what and relative to what? Just because training (or overfitting) plateaus after X epochs hardly means that the architecture is inherently stable. In fact, architectures involving adversarial networks are prone to mode collapse and infamously unstable. Furthermore, GANs are not broadly considered interpretable models. This “results” section calls into question the whole paper for me. In particular, training stability should not serve as a proxy for robustness that matters to scRNA-seq researchers (stability in light of biological or experimental variability). Furthermore, highlighting one gene without clearly describing how they performed feature-wise attribution does not convince me that their method is “interpretable”.

*We thank the reviewer for this concern. As we stated in the manuscript, “We further used the ‘panc_rm’ dataset to demonstrate the stability of iMAP under the effects of hyperparameters and stochasticity”, by stability, we mean that the selection of hyperparameters has relatively small effects on the performance (Supplementary Fig. S10) and when we run iMAP multiple times, the performance is stable (Figure 3d, e, f). The number of training epochs is also taken as an adjustable hyperparameter, and because we only have the losses as the guideline during the training, we regard the wide range of training epochs (between 50-150) with small fluctuations of losses as the indicator of stability over the specific selection of stopping point. We additionally tested and discussed the effects of network structures under a previous suggestion of the reviewer (Major comments 1) in the updated manuscript (Table S3, S4). As the reviewer pointed out, the robustness over biological and experimental variability is much more important than training stability. We demonstrated the robustness of iMAP by applying it to over 10 real-world datasets in the benchmark section of the manuscript (including “Benchmark evaluations” and “Integration of human pancreas datasets”).*

*The interpretability is still a major concern in the application of deep models on biological systems. Current interpretability is mostly focused on assigning an importance score for each gene to evaluate its effect on the final objective [3]. Most interpretability methods are developed for supervised classification problems since their objectives are apparent. For our problems, the objectives of batch effect removal can be formulated as accurately identifying the cell type identity and sufficiently removing the differences of batches. So, we assign two importance scores for each gene. To clearly illustrate how the gene-wise importance scores are calculated, we depict the details in the Supplementary Fig. S1c, d, and explain them in the ‘Methods’ section (‘Explanations of gene importance’). The cell type importance scores are obtained by fabricating one classification problem with the representations from the encoder E as inputs and the true cell type label as outputs. The batch importance scores are obtained by the other classification problem with the expression vectors as inputs (which are also the inputs of GAN), and the batch label as outputs. As we stated in the manuscript, we only regarded these as the surrogate for evaluating the importance of one gene in removing batch effects and primary heuristics, and did not pursue the complete explanation of the working mechanisms of GAN.*

Minor comments

1. The pre-processing of the human pancreas datasets seems likely to largely influence performance. I suggest doing a sensitivity analysis here where cell types are not removed from the dataset. Does iMAP still perform better than other methods in these more realistic scenarios? The results are hard to follow here so perhaps the authors did this but could clarify this section.

*We thank the reviewer for this question. We performed batch effect removal on the complete ‘panc’ dataset using iMAP and all other nine methods, with the results shown in Supplementary Fig. S2b. The performance of iMAP is better than others. We also included a citation in the main text:*

*“Particularly, we removed cells of two cell types with very large number of cells (i.e. ‘acinar’ and ‘alpha’ cells) from the ‘inDrop’ sub-dataset, and ‘ductal’ cells from the ‘CEL-seq’ sub-dataset (See Supplementary Fig.S2b for the complete ‘panc’ dataset; See Methods for the exclusion criteria).*

”

2. The section on large-scale datasets is not a fair comparison, necessarily, as methods such as BB-kNN show scalability of integration across orders of magnitude, rather than just 100,000 cells. A more fair comparison would be a discussion as to how iMAP has unique advantage on small/medium/large datasets but this is impossible to gauge with the current discussion because even though they downsample, they do not downsample and compare their method to others, which seems to be the key piece of insight of interest which is missing.

*We thank the reviewer for this comment. Previously, we just emphasized the scalability of iMAP and did not perform comparison with other methods, because the time cost of iMAP is largely dependent on the GPUs, while such cost of methods including BBKNN is dependent on the performance of CPUs. We included a comparison with others in the new Supplementary Fig. S12c, and when cell number is equal to 100,000, the time costs of iMAP, fastMNN, and BBKNN are comparable, and much lower than others. In particular, we would like to highlight that iMAP is much faster than other deep learning-based models, such as scVI and DESC.*

3. There is poor justification for throwing together an autoencoder with a GAN that could be better explored. Interpolation in an autoencoder’s latent space is typically meaningless, so it is unclear why it is necessary to use an autoencoder for preserving biological content, when an appropriately trained or constrained GAN can (and has for single-cell data) generate realistic single-cell data, conditional on some label. Can the authors more systematically explore hyperparameters and ablation studies with various autoencoder and GAN architectures? Or, for example, how do low rank approximations compare to the encoder representation?

*We thank the reviewer for this question. Our autoencoder structures construct latent representations of single cells, which we expect to have at least two kinds of properties, with the first to preserve the biological contents, and the second to roughly remove the batch effects. This is also the rationale behind the content loss. As the reviewer suggested in the first major comment, we explored more ablation studies to confirm the necessity of our autoencoder and GAN architectures. As shown in Table S4, substituting the autoencoder with low-rank approximations, such as PCA, or substituting the GAN with the original MNN-based correction, would significantly reduce the performance.*

4. Nhu Tran et al. 2020 propose five evaluation metrics to compare batch-effect correction methods. Why not do the same and use a similar dataset to frame iMAP?

*We thank the reviewer for this suggestion. We only considered single-cell level evaluation metrics, such as LISI and our proposed novel metrics in our original manuscript, because as shown in Supplementary Fig. S1a, cluster-level metrics, such as ASW and ARI used in [4], cannot accurately evaluate the local integration of cells from different batches. We added the kBET metric in the updated manuscript as stated in a previous question (Major comments 2). We tested almost all datasets of [4], and added some other datasets as shown in Table S1. Since some cell type labels were obtained by computational methods, we think that it is not necessary to conduct an exhaustive comparison. As stated in a previous question (Major comments 2), two additional complicated datasets were included in the updated benchmark, and we hope this is adequate to demonstrate the preeminent performance of iMAP.*

Reviewer #2: The authors have done a great job addressing my previous concerns. I don't have any additional comments.

*We thank the reviewer for the positive comments on our work.*

*References*

*1.	Haghverdi L, Lun ATL, Morgan MD, Marioni JC: Batch effects in single-cell RNA-sequencing data are corrected by matching mutual nearest neighbors. Nat Biotechnology 2018, 36:421-427.*

*2.	Ding J, Adiconis X, Simmons SK, Kowalczyk MS, Hession CC, Marjanovic ND, Hughes TK, Wadsworth MH, Burks T, Nguyen LT, et al: Systematic comparison of single-cell and single-nucleus RNA-sequencing methods. Nat Biotechnology 2020, 38:737-746.*

*3.	Zhou Z, Ye C, Wang J, Zhang NR: Surface protein imputation from single cell transcriptomes by deep neural networks. Nat Communications 2020, 11:651.*

*4.	Tran HTN, Ang KS, Chevrier M, Zhang X, Lee NYS, Goh M, Chen J: A benchmark of batch-effect correction methods for single-cell RNA sequencing data. Genome Biology 2020, 21:12.*

**Third round of review**

**Reviewer 1**

The authors of iMAP have attempted to address all of my comments by providing ablation studies, more comparisons with benchmarks, evaluating performance across a small hyperparameter space, and increasing the number of used data sets to examine scalability. I still have some minor concerns. For example, there are modest modifications to depth and width tested on data sets are all on the order of 1,000 - 10,000 cells, leaving open the question of scalability w.r.t. guiding neural architecture search for larger data sets. In table S4, the numbers of cells seems to differ greatly across trials, presumably because they only report true negative numbers and the false positive rate is high in some of their trials? Nonetheless, the results show that iMAP, in it's full form, is justified, which was my concern. I still have some concern about the reader's potential mis-interpretation of the "interpretability" section of the GAN, so the authors could consider tempering their statements or signaling limitations of this in the discussion but this is a minor point.
